# Supplementary material for: Latent Style-Based Quantum Wasserstein GAN for Drug Design
Source: J Chem Inf Model. 2026 Oct 13;66(20):13136–54. doi: 10.1021/acs.jcim.6c01249 (PMC13715521; doi:10.1021/acs.jcim.6c01249)
Supplement: Supplementary file 1 [file ci6c01249_si_001.pdf]

# Supporting Information

## Latent Style-based Quantum Wasserstein GAN for Drug Design

Julien Baglio [1,2,*], Yacine Haddad [3,4,*] and Richard Polifka [2]

[1]Center for Quantum Computing and Quantum Coherence (QC2), Department of Physics, University of Basel, Klingelbergstrasse 82, CH-4056 Basel, Switzerland
[2]QuantumBasel, Schorenweg 44b, CH-4144 Arlesheim, Switzerland
[3]Laboratory for High Energy Physics (LHEP), Physics Institute, Faculty of Sciences, University of Bern, Sidlerstrasse 5, CH-3012 Bern, Switzerland
[4] European Organization for Nuclear Research (CERN), Espl. des Particules, CH-1211 Geneva 23, Switzerland

[*]Corresponding authors: julien.baglio@unibas.ch; yacine.haddad@unibe.ch

This Supporting Information accompanies the article published at https://doi.org/10.1021/acs.jcim.6c01249. It contains the following: distributions of the input train and test datasets; a study of the number of epochs for the VAE, including the loss function as a function of the number of epochs, the various metrics distributions, and a table reporting the metrics and GPU runtime as a function of the number of epochs; a metrics comparison for the VAE when generating more than 30,000 new molecules; details of the VAE encoder and decoder hyperparameter tuning, including figures and tables; a detailed study of the VAE decoder confirming its correct performance; a study of the correlations between the components of the latent vectors, including the correlation matrices; detailed results for all the QGAN scenarios investigated, including tables and distribution plots, for latent dimensions from 10 to 30 and from five to 15 qubits; a classical GAN control study with generator capacities matched to the QGAN order of magnitude (682, 346, 115, 110, and 94 trainable parameters), including loss-function and metrics comparisons; and, for the illustrative IBM inference run described in the main text, quantum hardware characterization including execution metadata and transpiled-circuit statistics from job records, representative device calibration properties from execution-date ibm_kingston backend properties, per-metric $Z_0$ bootstrap analysis of the hardware run versus the noiseless simulation, and a matched `FakeKingston` noise-model simulation comparing noiseless simulation, noisy simulation, and hardware molecular metrics, together with a direct comparison of pre-VAE latent-vector distributions across the three inference settings.

# 1 SUPPLEMENTARY MATERIAL: INPUT DISTRIBUTIONS

In this section, the five main metrics are presented, calculated from the input training and test datasets. The plots are in Figure 1 and show certain differences between the two distributions, except for the LogP metric.

Table 1 then shows the mean internal diversity and the Wasserstein distance from the test sample.

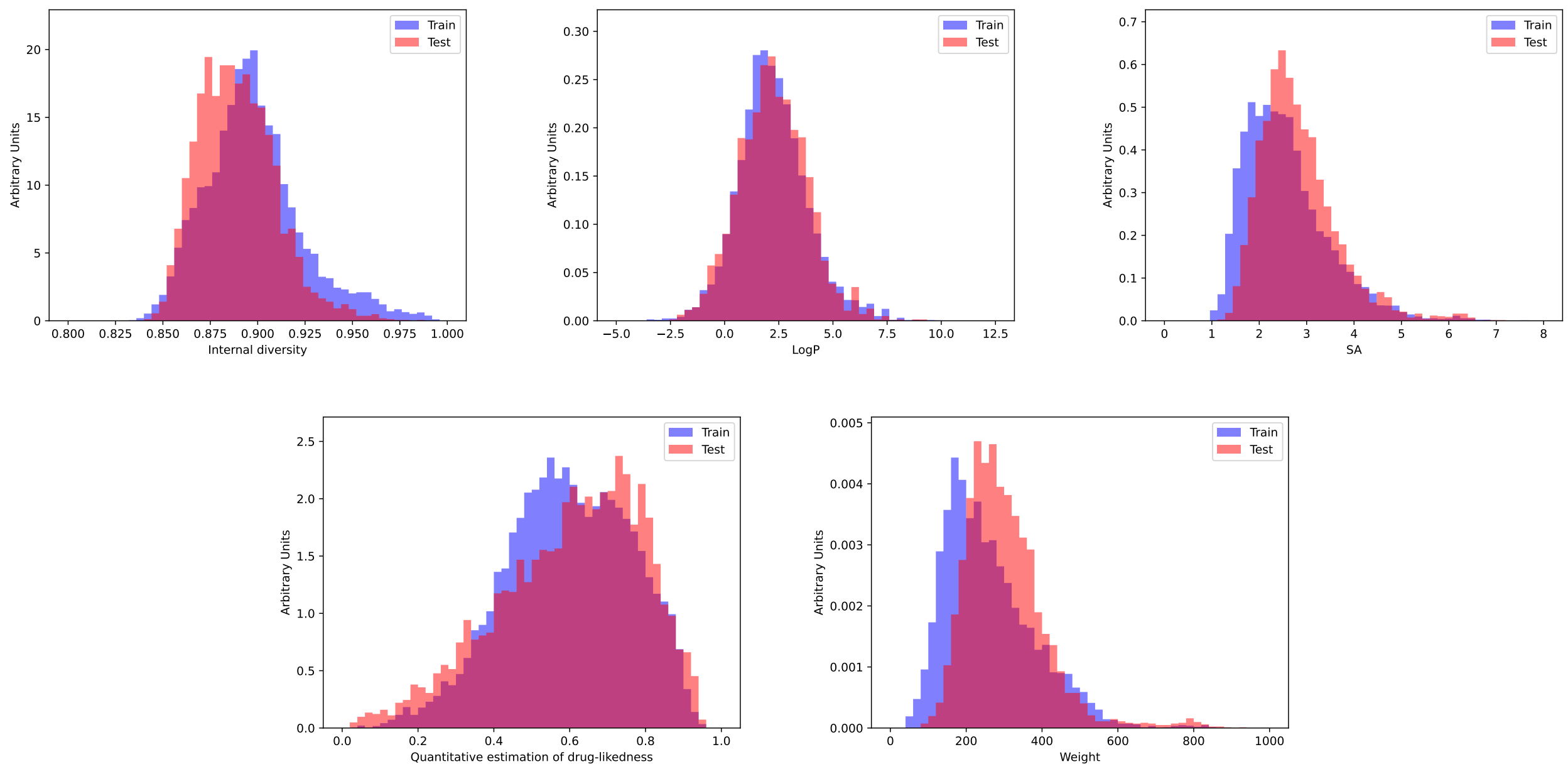


**Figure 1.** Comparison of metrics distributions between the input train and test sets.

| Metrics | Train set | Test set |
|---|---|---|
| IntDiv | 0.898 | 0.889 |
| $\mathcal{W}$(LogP) | 0.091 | – |
| $\mathcal{W}$(SA) | 0.268 | – |
| $\mathcal{W}$(QED) | 0.027 | – |
| $\mathcal{W}$(Weight) | 42.8 | – |

**Table 1.** Comparison of metrics between the input train and test sets.

# 2 SUPPLEMENTARY MATERIAL: VAE STUDY OF $N_{ep}$

To ensure stable model training, the sensitivity of the loss function and metrics to the number of training epochs has to be studied. The general rule is that the loss function around the final chosen point of $N_{ep}$ should be stable for the training as well as the test dataset.

Figure 2 presents the dependency of the loss function for both sets as a function of the number of training epochs. From the requirement of stability, it follows that anything below $N_{ep} = 800$ might not be stable enough. Four working points have been chosen to test the hypothesis:

1. $N_{ep} = 100$: default value from the MOSES library;
2. $N_{ep} = 250$: value where the training set seems to be stable but the test set loss function is still increasing;

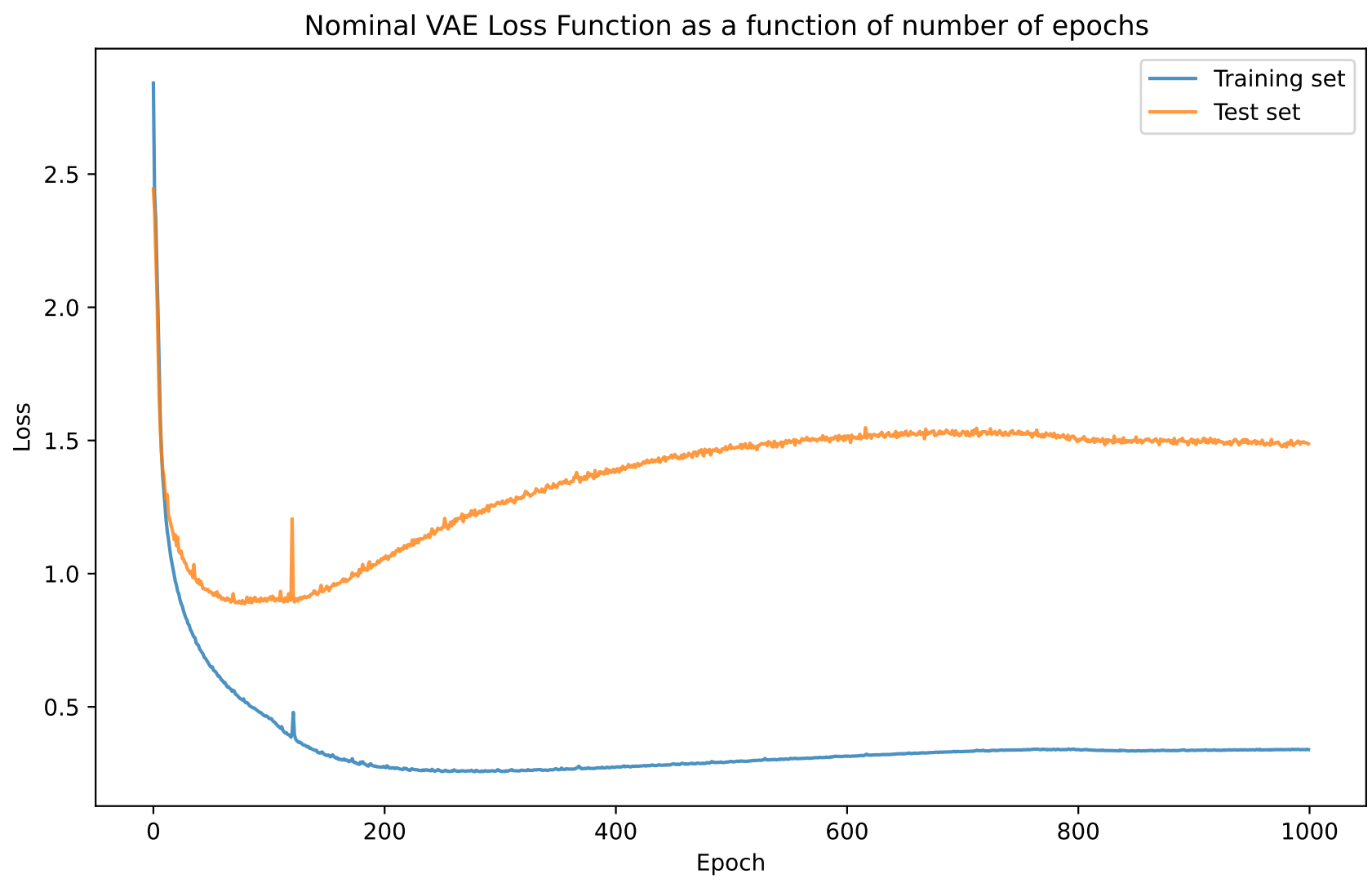


**Figure 2.** VAE loss function as a function of number of training epochs for the nominal VAE.

3. $N_{ep} = 800$: value where both loss functions seem to start to be stable;
4. $N_{ep} = 1000$: value chosen to be far from the beginning of the stable period of both loss functions.

The trainings have been performed for the four amounts of epochs, and the results are summarized in Table 2. As expected, the values for the working point of 800 and 1,000 are consistent, hence supporting the theory that stable loss functions mean stable metric results and justifying the final choice of $N_{ep} = 1,000$ for the VAE training. The same conclusion can be drawn from Figure 3 where an agreement is observed for the 800 and 1,000 working points while (in particular for internal diversity and molecular weight) the results for the 100 and 250 working points diverge.

| $N_{ep}$ | 100 | 250 | 800 | 1,000 |
|---|---|---|---|---|
| GPU runtime | 9m 10s | 17m 24s | 48m 49s | 52m 35s |
| IntDiv | 0.892 | 0.896 | 0.895 | 0.896 |
| $\mathcal{W}$(LogP) | 0.190 | 0.086 | 0.088 | 0.087 |
| $\mathcal{W}$(SA) | 0.285 | 0.265 | 0.280 | 0.275 |
| $\mathcal{W}$(QED) | 0.025 | 0.023 | 0.023 | 0.025 |
| $\mathcal{W}$(Weight) | 67.6 | 51.2 | 43.8 | 43.9 |

**Table 2.** VAE metrics comparison for the four $N_{ep}$ points. The Wasserstein distance is measured between the test and generated samples. While the internal diversity is maximized, all other metrics are minimized. We also report the GPU runtime for comparison.

## 3 SUPPLEMENTARY MATERIAL: DEPENDENCY ON $N_{Gen}$

This section investigates the question of whether 30,000 samples generated for the purpose of validating the molecular sets are sufficient.

Once the sampler is called and generates the requested amount, a three-step efficiency estimation (as described in Section 2.3 of the main text) is performed. The number of relevant molecules that are used to derive all the metrics is defined in eq 1 as:

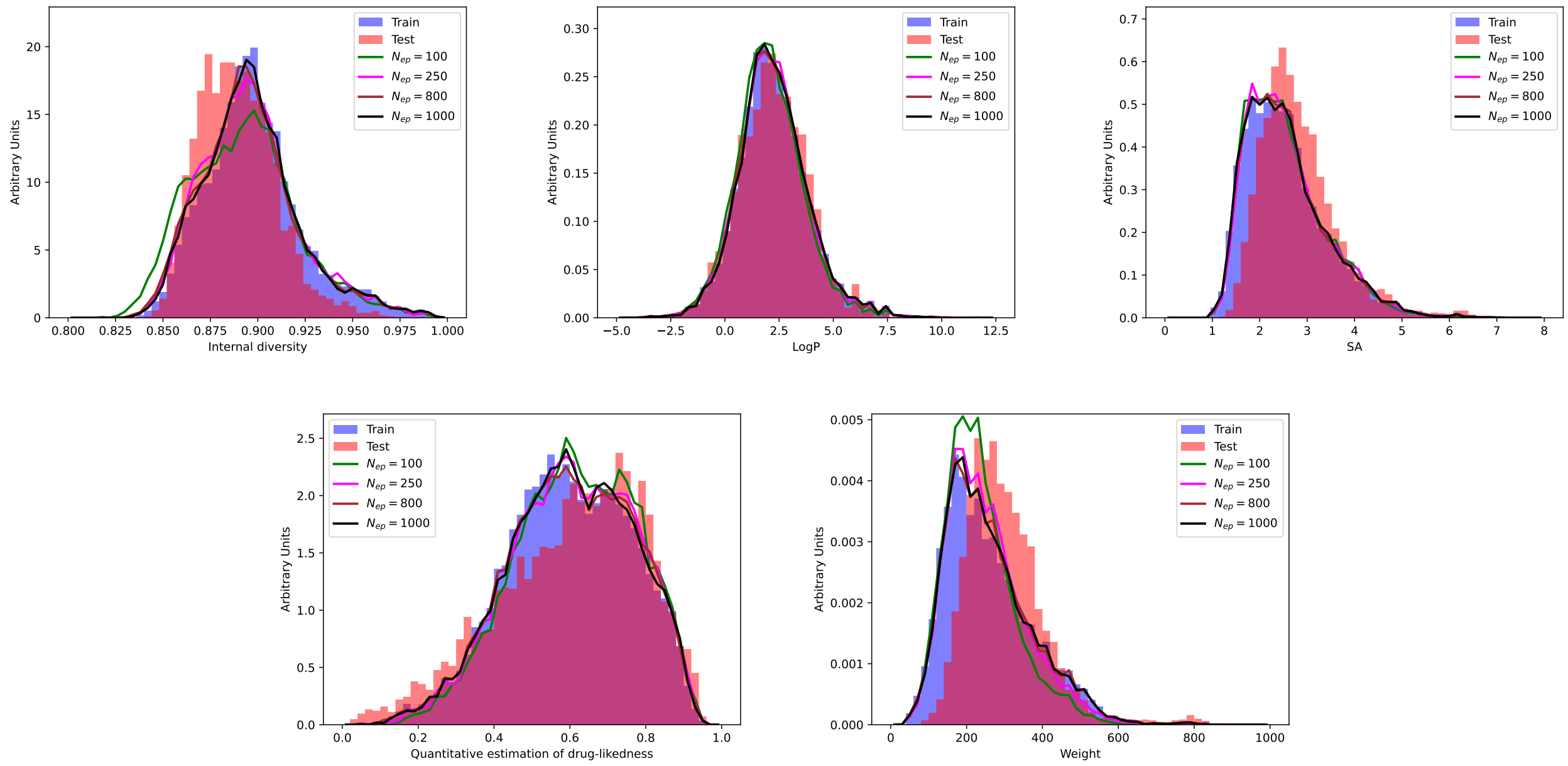


**Figure 3.** Metrics distributions for models trained with different number of epochs. Stabilization of the results starts with 800 epochs.

$$N_{Canonical} = N_{Generated} \times \epsilon_d \times \epsilon_v \times \epsilon_u \qquad (1)$$

where $\epsilon_d$ quantifies the amount of identically duplicated strings, $\epsilon_v$ the amount of chemically valid molecules, and $\epsilon_u$ the amount of canonically distinct molecules.

Table 3 presents the results for the nominal VAE settings and three different sizes of the generated sample. The trend of increasing inefficiency is clearly visible: while the amount of identical strings for the sample with 30,000 molecules (the 30k sample) is almost 50%, for the 1M set it is almost 90%. While the fraction of unique molecules stays almost constant, the fraction of valid molecules also drops significantly with increasing number of generated samples. The combined effect is then visible in the number of canonical generated molecules, which, for a 33-fold increase in initial statistics, grew only by a factor of six.

Worth mentioning is also the observed trend in the novelty, which increases dramatically. However, this increase does not immediately mean an increased capacity to generate new molecules. The novelty is defined as the number of generated molecules that were not present in the training sample. Although this holds well for the original MOSES analysis with more than 1M molecules used for training, it is important to keep in mind that this analysis used only 12,000 molecules for training. That means that even if all training samples would be present in the 1M set, the novelty would be 83%. Same calculation for the 30k sample yields such minimal novelty of 6%. Comparing with the results from Table 3, we see that the 30k sample actually generates more new molecules than the 1M sample. The 30k scenario, resulting in about 13,000 valid canonical molecules, is therefore a good candidate for a reliable novelty calculation in an analysis where only 12,000 training molecules are used.

All these arguments, together with minimizing the sampling time, lead to the decision of using 30,000 generated samples for subsequent result estimation.

## 4 SUPPLEMENTARY MATERIAL: VAE HYPERPARAMETER TUNING

The tuning of the VAE was performed for three distinct domains: the learning rate, the encoder parameters, and the decoder parameters. Tables 4, 5 and 6 present the parameter variations as well as the metrics comparison.

| $N_{generated}$ | **30,000** | **300,000** | **1,000,000** |
|---|---|---|---|
| GPU runtime | 52m | 1h 37m | 2h 12m |
| Sampling runtime | 10m | 55m | 1h 30m |
| $\epsilon_d$ | 0.479 | 0.168 | 0.120 |
| $\epsilon_v$ | 0.889 | 0.704 | 0.609 |
| $\epsilon_u$ | 0.996 | 0.982 | 0.977 |
| $N_{Canonical}$ | 12,725 | 34,763 | 71,118 |
| Novelty | 0.237 | 0.662 | 0.835 |
| IntDiv | 0.896 | 0.896 | 0.895 |
| $\mathcal{W}$(LogP) | 0.087 | 0.066 | 0.094 |
| $\mathcal{W}$(SA) | 0.275 | 0.188 | 0.127 |
| $\mathcal{W}$(QED) | 0.025 | 0.020 | 0.017 |
| $\mathcal{W}$(Weight) | 43.9 | 38.4 | 32.6 |

**Table 3.** Metrics comparison for the nominal VAE training for three different sizes of sets of generated molecules. We also report runtime on the GPU.

| lr max | $3 \times 10^{-4}$ | $1 \times 10^{-4}$ | $1 \times 10^{-2}$ | $5 \times 10^{-5}$ | $5 \times 10^{-3}$ | $1 \times 10^{-3}$ |
|---|---|---|---|---|---|---|
| lr min | $3 \times 10^{-4}$ | $1 \times 10^{-6}$ | $1 \times 10^{-4}$ | $5 \times 10^{-5}$ | $5 \times 10^{-3}$ | $1 \times 10^{-5}$ |
| $\epsilon_d$ | 0.479 | 0.805 | 0.938 | 0.842 | 0.936 | 0.417 |
| $\epsilon_v$ | 0.889 | 0.660 | 0.018 | 0.626 | 0.041 | 0.957 |
| $\epsilon_u$ | 0.995 | 0.986 | 0.956 | 0.985 | 0.940 | 0.997 |
| Novelty | 0.237 | 0.702 | 0.925 | 0.768 | 0.856 | 0.111 |
| IntDiv | 0.896 | 0.893 | 0.887 | 0.891 | 0.929 | 0.898 |
| $\mathcal{W}$(LogP) | 0.087 | 0.104 | 13.789 | 0.133 | 0.823 | 0.083 |
| $\mathcal{W}$(SA) | 0.275 | 0.292 | 1.731 | 0.305 | 0.398 | 0.265 |
| $\mathcal{W}$(QED) | 0.025 | 0.026 | 0.394 | 0.025 | 0.131 | 0.026 |
| $\mathcal{W}$(Weight) | 43.9 | 57.3 | 1261.2 | 62.3 | 169.9 | 43.1 |

**Table 4.** Comparisons of the results of VAE learning rate tuning, the first column corresponds to the nominal scenario. Individual scenarios are defined by the learning rate minima/maxima at the top of the Table.

Table 4 and Figure 4 present the results of the learning rate optimization. It is obvious from the plots that, when the learning rate is too large (0.01-0.0001 and 0.005 scenarios), the results collapse, and the metrics become meaningless, as reflected in Table 4. Comparison of the remaining scenarios prefers the 0.001-0.00001 tuning.

Table 5 and Figure 5 present the results of the encoder optimization. Although there seems to be a slight inclination to increase the hidden dimension to 512, changing the number of layers to two does not provide any improvement.

Table 6 and Figure 6 present the results of the decoder optimization. Unlike the encoder, there seems to be a preference for increasing the dropout to 0.75 and the number of layers to four, while the hidden layer dimension stays the same.

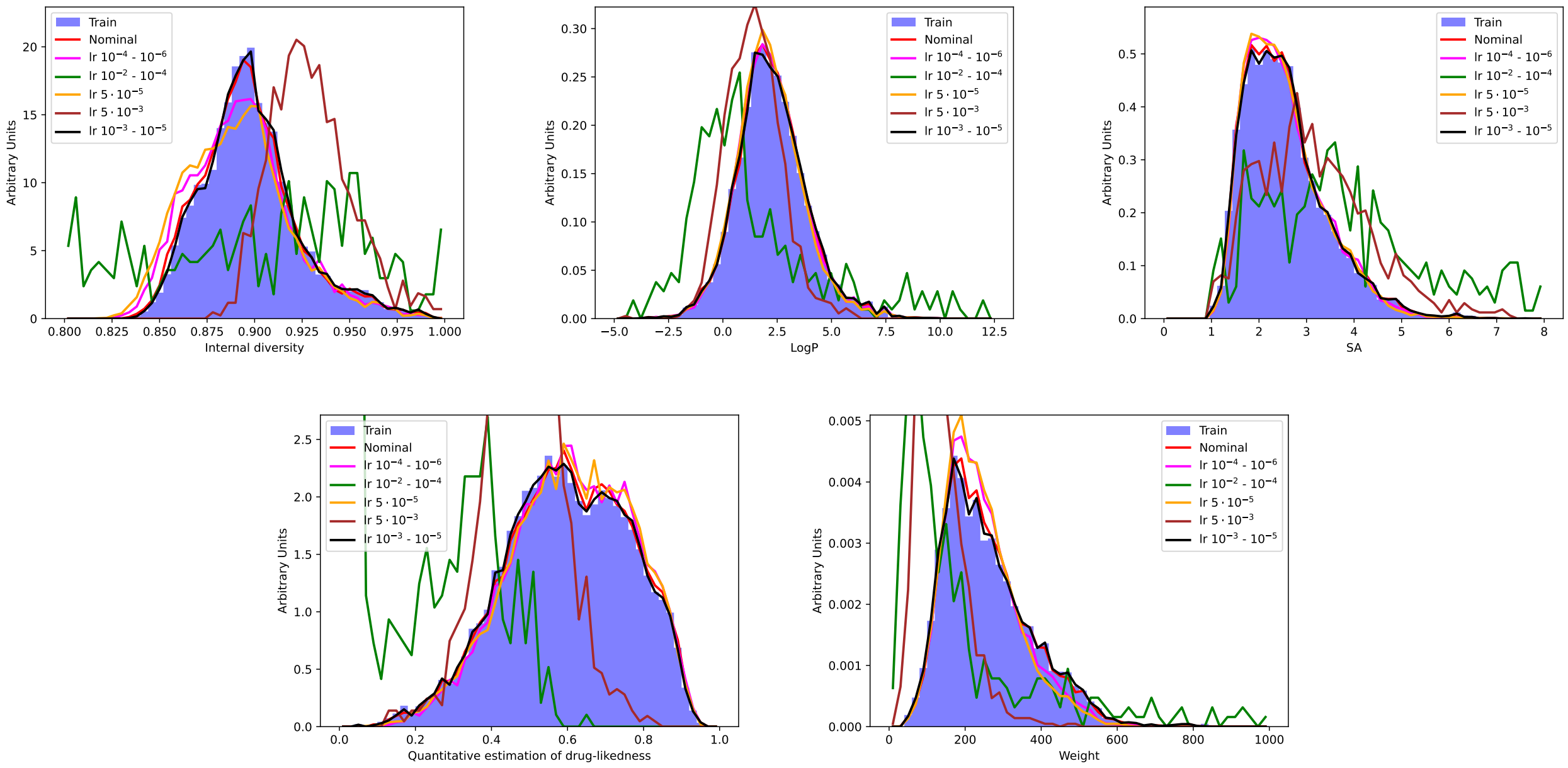


**Figure 4.** Metrics distributions for models trained with different learning rates. Clear breakdown for the 0.01-0.0001 and 0.005 scenarios is visible.

| | | | | |
|---|---|---|---|---|
| $n_l$ | 1 | **2** | 1 | 1 |
| $D_h$ | 256 | 256 | **128** | **512** |
| Dropout | 0.0 | **0.5** | 0.0 | 0.0 |
| $\epsilon_d$ | 0.479 | 0.548 | 0.475 | 0.5321 |
| $\epsilon_v$ | 0.889 | 0.838 | 0.888 | 0.8552 |
| $\epsilon_u$ | 0.996 | 0.994 | 0.995 | 0.9933 |
| Novelty | 0.237 | 0.349 | 0.238 | 0.327 |
| IntDiv | 0.896 | 0.895 | 0.896 | 0.896 |
| $\mathcal{W}$(LogP) | 0.087 | 0.094 | 0.075 | 0.087 |
| $\mathcal{W}$(SA) | 0.275 | 0.273 | 0.271 | 0.269 |
| $\mathcal{W}$(QED) | 0.025 | 0.024 | 0.026 | 0.025 |
| $\mathcal{W}$(Weight) | 43.9 | 44.3 | 43.6 | 45.3 |

**Table 5.** Comparisons of the results of VAE encoder parameter tuning, the first column corresponds to the nominal scenario. The scenario variation is highlighted at the top of the table. The dropout is active only for a scenario with more than one encoder layer, in such case value of 0.5 was used.

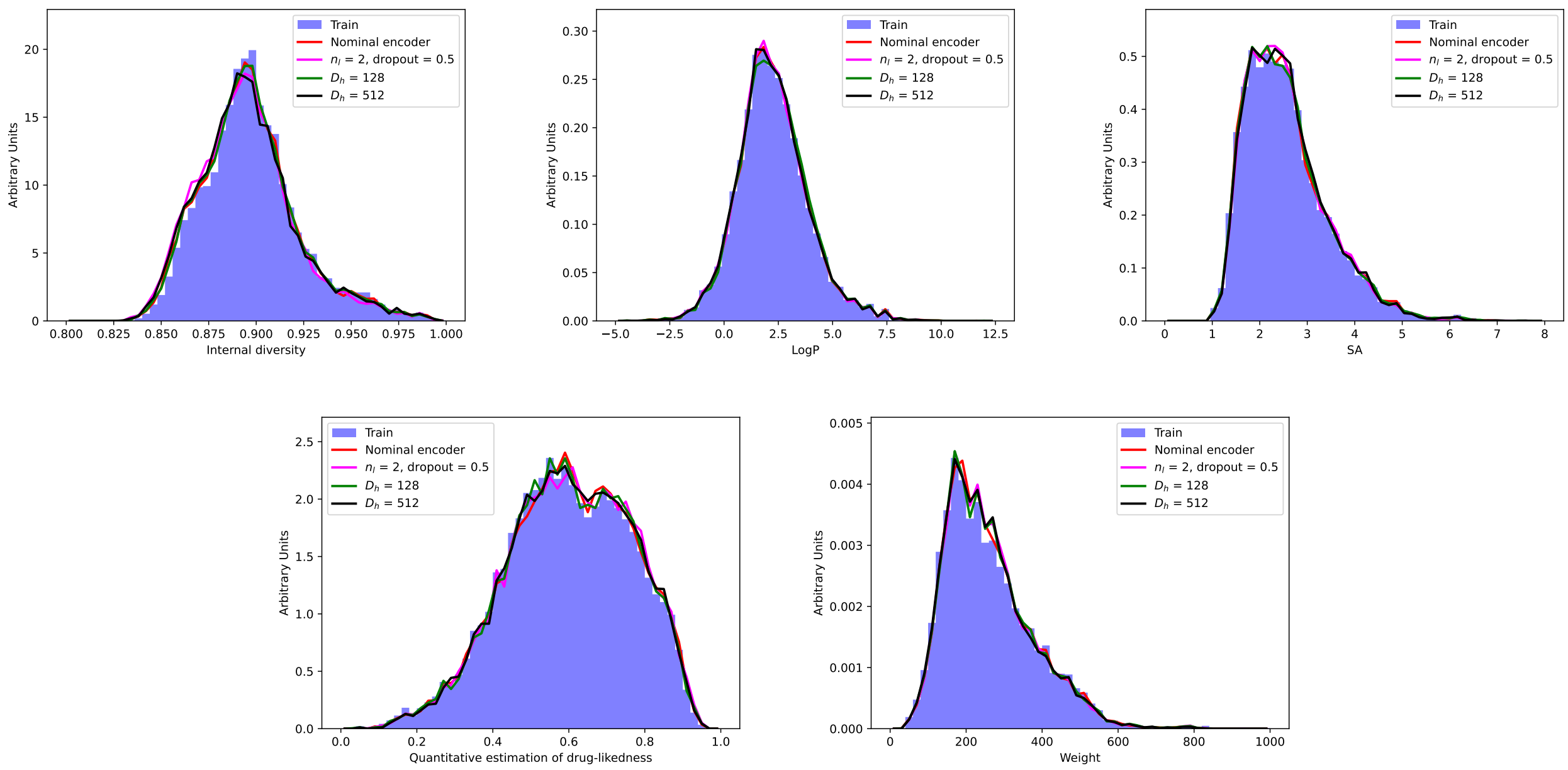


**Figure 5.** Metrics distributions for models trained with different encoder settings. Results are very similar across different scenarios.

| | | | | | | | |
|---|---|---|---|---|---|---|---|
| $n_l$ | 3 | **2** | **4** | 3 | 3 | 3 | 3 |
| $D_h$ | 512 | 512 | 512 | **256** | **1024** | 512 | 512 |
| Dropout | 0.0 | 0.0 | 0.0 | 0.0 | 0.0 | **0.25** | **0.75** |
| $\epsilon_d$ | 0.479 | 0.590 | 0.487 | 0.718 | 0.416 | 0.480 | 0.556 |
| $\epsilon_v$ | 0.889 | 0.818 | 0.869 | 0.718 | 0.944 | 0.911 | 0.875 |
| $\epsilon_v$ | 0.996 | 0.990 | 0.996 | 0.988 | 0.997 | 0.996 | 0.994 |
| Novelty | 0.237 | 0.429 | 0.240 | 0.573 | 0.126 | 0.248 | 0.378 |
| IntDiv | 0.896 | 0.896 | 0.897 | 0.894 | 0.898 | 0.897 | 0.896 |
| $\mathcal{W}$(LogP) | 0.087 | 0.098 | 0.088 | 0.096 | 0.087 | 0.084 | 0.081 |
| $\mathcal{W}$(SA) | 0.275 | 0.244 | 0.252 | 0.289 | 0.268 | 0.250 | 0.270 |
| $\mathcal{W}$(QED) | 0.025 | 0.025 | 0.025 | 0.023 | 0.027 | 0.025 | 0.023 |
| $\mathcal{W}$(Weight) | 43.9 | 48.4 | 41.7 | 51.3 | 45.3 | 42.6 | 43.6 |

**Table 6.** Comparisons of the results of VAE decoder parameter tuning, the first column corresponds to the nominal scenario. The scenario variation is highlighted at the top of the table. The default decoder dropout is zero regardless the number of layers.

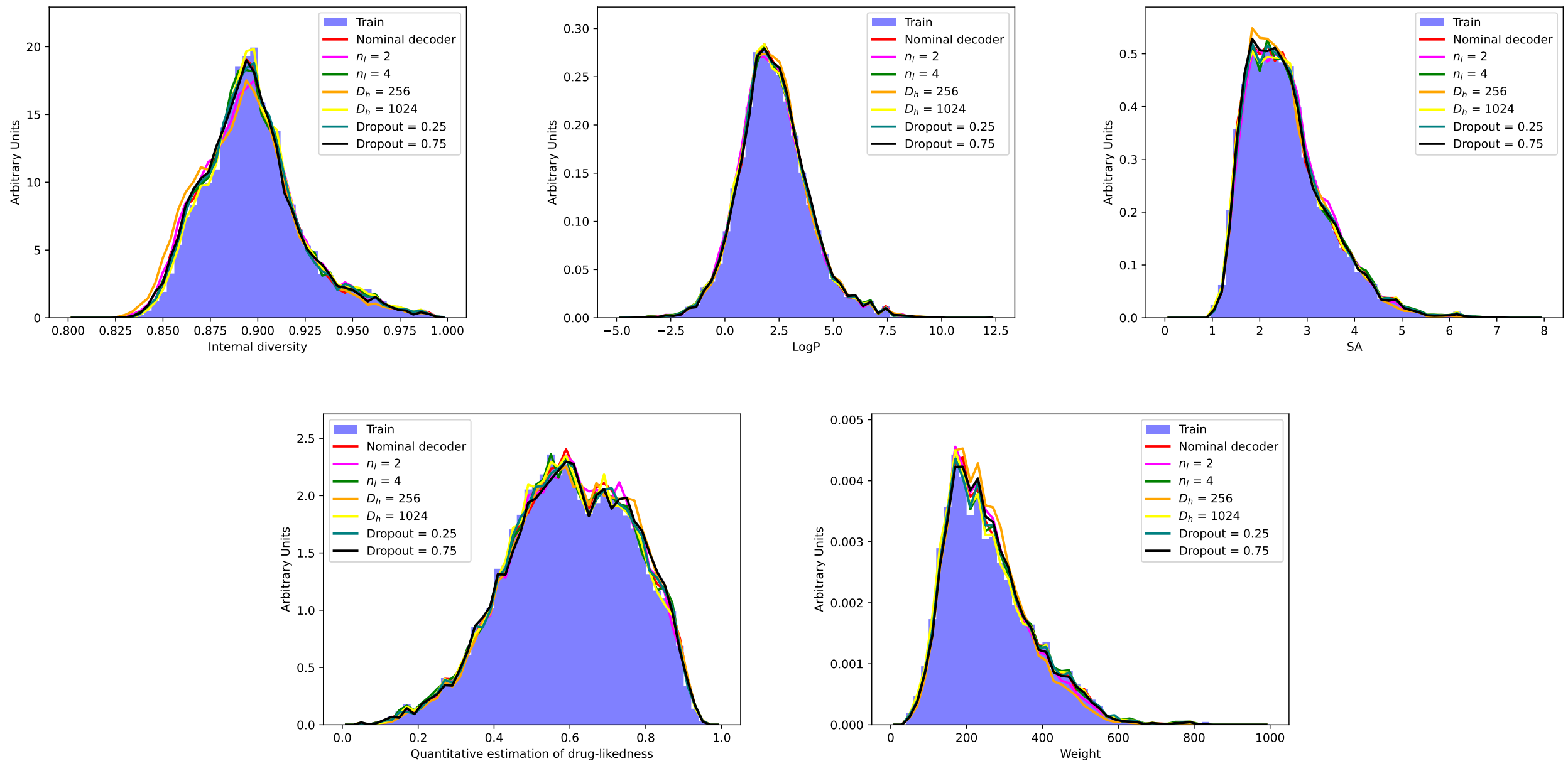


**Figure 6.** Metrics distributions for models trained with different decoder settings. Results are very similar across different scenarios.

## 5 SUPPLEMENTARY MATERIAL: TEST OF THE VAE DECODER

In order to be able to claim that the VAE is functioning properly, it is important to test whether its decoding part is able to distinguish between different inputs. In other words, whether the decoder is sensitive to the shape of the distributions of the latent vectors or whether any distribution collapses to the same output.

The base latent space of dimension 10 was used, and numbers drawn from the following distributions were decoded and processed using the standard metric evaluation:

1. The analysis training sample of 12,000 SMILES strings encoded by the VAE;
2. The analysis test sample of 4,087 SMILES strings encoded by the VAE;
3. 10,000 samples drawn from a uniform distribution between 0.0 and 1.0;
4. 10,000 samples drawn from a normal distribution with $\mu = 0.0$ and $\sigma = 1.0$;
5. 10,000 samples drawn from a log-normal distribution with $\mu = 0.0$ and $\sigma = 0.558$ shifted by two units to the negative values;
6. 10,000 samples drawn from a $sin$ distribution with three peaks in the range $[-3; 3]$.

The distributions of the 10 vectors are presented in Figure 7, clearly showing the different shapes. The first two samples corresponding to the train and test encoded datasets exhibit similar behavior consistent with the normal(0;1) distribution, except for vectors number six and seven.

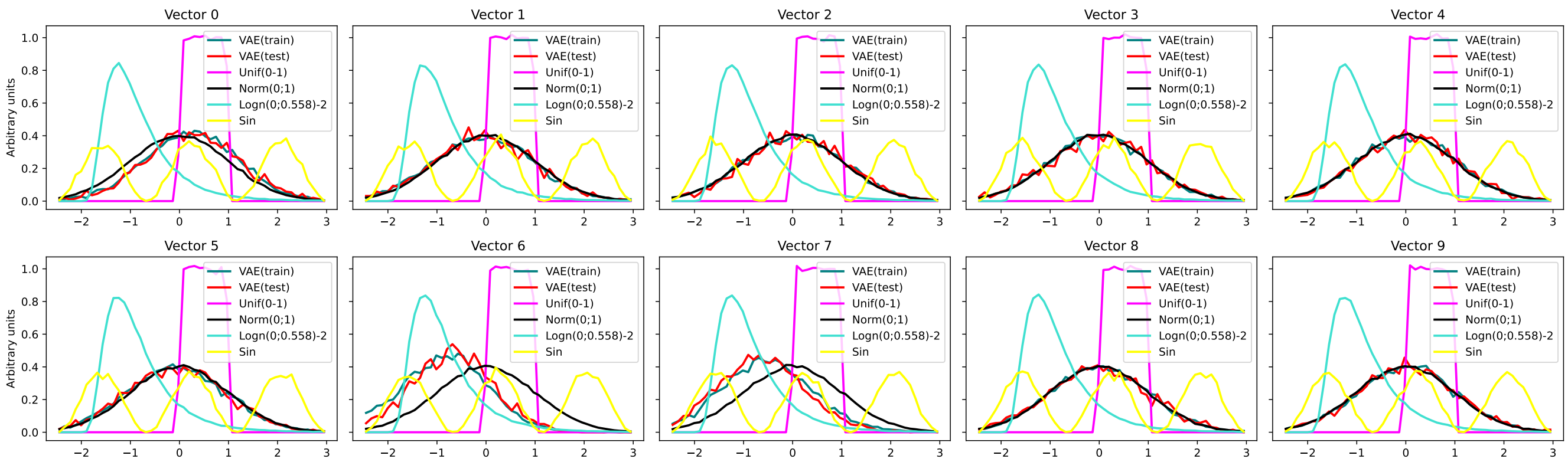


**Figure 7.** Distributions of 10 latent vectors used as inputs to the VAE decoder.

The sets of SMILES strings produced by the VAE decoder were subsequently evaluated in order to obtain the metrics. The comparison is in Figure 8.

The two shaded histograms correspond to the initial train and test samples without any use of VAE and clearly exhibit distinct behavior. However, the same samples, when encoded and decoded by the VAE (red and green), end up very consistent with each other but not with either the original train or test samples. This might hint at some level of information loss in the process of encoding and decoding the sample through a latent space of dimension 10 only.

The VAE(gen) is very similar to the distribution originating from the uniform sampling in the latent space (brown and black). This is not very surprising because the internal MOSES VAE routine uses a uniform distribution as input for the sampler.

The uniform distribution has the most extreme shape difference with respect to the others (flat vs peak-y structure) and also shows the largest deviations in the metric distributions. The VAE decoder seems to treat all distributions that exhibit a peak structure in a similar way.

Surprisingly, the metrics distribution originating in the log-normal scenario seems to be the most similar to the VAE(test) and VAE(gen) samples. That might be explained by the fact that the input distributions for vectors six and seven seem to be more consistent for the VAE(train) and VAE(test) with the log-normal

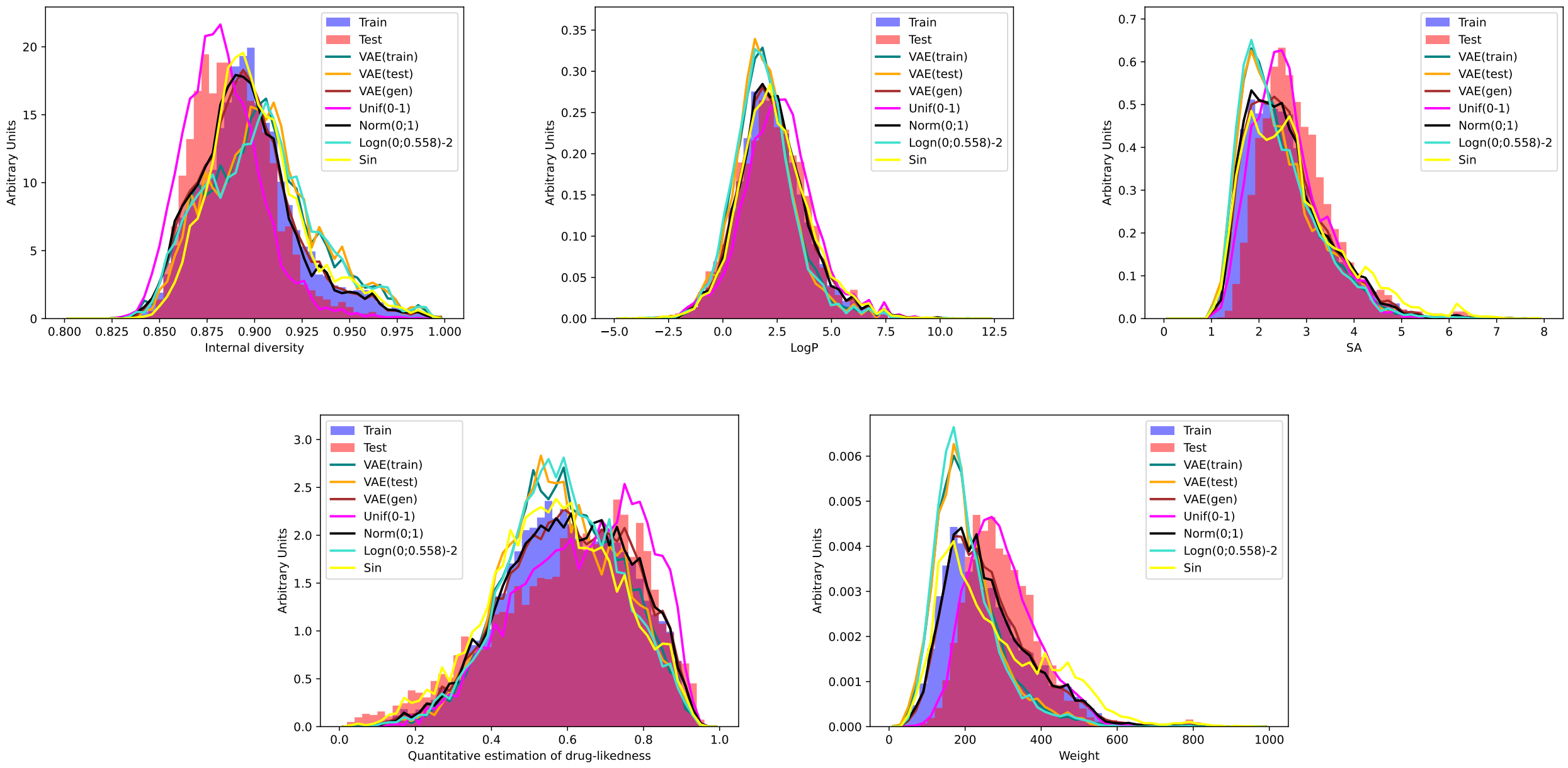


**Figure 8.** Metric distribution obtained from SMILES sets which were generated by the VAE decoder from various latent vector distributions. Additional scenario – VAE(gen) – corresponds to a set which is generated by the standard MOSES sampling procedure. The distributions are normalized to unit area.

rather than normal distributions. That would also hint that these two vectors are the ones with the largest discriminating power.

However, one can conclude that the VAE decoder is working as intended and is capable of producing SMILES sets with different feature characteristics depending on the shape of the latent vectors.

## 6 SUPPLEMENTARY MATERIAL: CORRELATIONS BETWEEN LATENT VECTORS

To complement the study of the latent space of dimension 10, this section presents the correlation matrices for the train, test, classical GAN, and QGAN datasets. While the train and test latent samples are simply a transformation of the SMILES sets through the VAE encoder, the samples for the GANs stem from the respective generators.

Figure 9 presents the correlation matrices. All vectors apart from numbers six and seven are almost perfectly uncorrelated, while the numbers six and seven show a small level of anti-correlation. This effect has different sizes in the four samples and is manifested by differing distributions for the respective vectors as presented in Figure 9 of the main text.

## 7 SUPPLEMENTARY MATERIAL: SUPPORTING MATERIAL FOR QGAN SCENARIOS

In this section, all detailed results supporting summary Table 5 of the main text are presented. Each of the following scenarios is represented by the corresponding metrics table and set of plots, for easy orientation, see Table 7.

All detailed results support the conclusion from Section 3.2 of the main text. Tables 12 and 13 use the GAN with the corresponding latent dimension for comparison with the QGANs. The average statistical significance is well below one standard deviation and hence statistically compatible with the results from the scenario comparison in Table 5 of the main text.

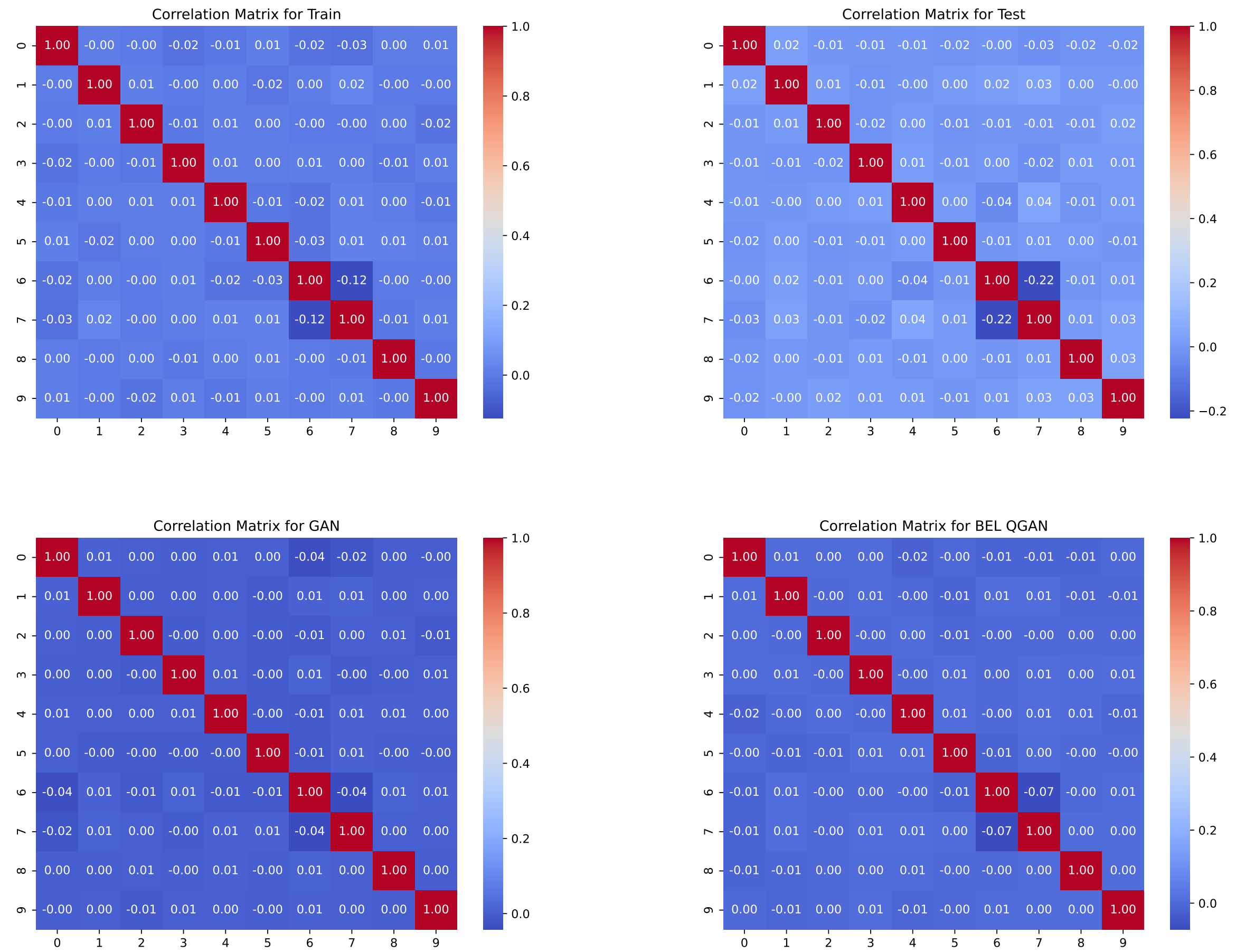


**Figure 9.** Correlations between individual latent vectors for the training set, test set, classical GAN training output, and BEL QGAN training output, all for a latent dimension of 10.

| Latent dimension | QGAN | | | Table | Figures |
|---|---|---|---|---|---|
| | $n_{qb}$ | $n_l$ | Readout | | |
| 10 | 5 | 2 | Dual | 8 | 8 of the main text |
| 10 | 5 | 4 | Dual | 9 | 10 |
| 10 | 10 | 2 | Single | 10 | 11 |
| 10 | 10 | 4, 6 | Single | 11 | 12 |
| 20 | 10 | 2 | Dual | 12 | 13 |
| 30 | 15 | 2 | Dual | 13 | 14 |
| 10, 20, 30 | – | – | – | 14 | 15 |

**Table 7.** Summary of the different scenarios and links to the corresponding individual tables and figures.

| Metrics | Classical tuned | Styled simple QGAN | $Z_0$ | styled BEL QGAN | $Z_0$ |
|---|---|---|---|---|---|
| $N_{params}$ | 705,162 | 20 | | 110 | |
| $\epsilon_d$ | $0.481 \pm 0.031$ | $0.529 \pm 0.003$ | $+1.56$ | $0.523 \pm 0.003$ | $+1.36$ |
| $\epsilon_v$ | $0.917 \pm 0.012$ | $0.831 \pm 0.002$ | $-7.21$ | $0.875 \pm 0.003$ | $-3.43$ |
| $\epsilon_u$ | $0.986 \pm 0.003$ | $0.994 \pm 0.000$ | $+3.50$ | $0.993 \pm 0.001$ | $+2.90$ |
| Novelty | $0.536 \pm 0.015$ | $0.572 \pm 0.003$ | $+2.40$ | $0.548 \pm 0.003$ | $+0.82$ |
| IntDiv | $0.898 \pm 0.004$ | $0.882 \pm 0.000$ | $-4.60$ | $0.883 \pm 0.000$ | $-4.21$ |
| Filters | $0.721 \pm 0.011$ | $0.737 \pm 0.003$ | $+1.43$ | $0.719 \pm 0.002$ | $-0.17$ |
| $\epsilon_{LogP}$ | $0.898 \pm 0.007$ | $0.883 \pm 0.001$ | $-2.35$ | $0.896 \pm 0.002$ | $-0.23$ |
| $\langle$SA$\rangle$ | $2.391 \pm 0.080$ | $2.575 \pm 0.007$ | $-2.31$ | $2.448 \pm 0.007$ | $-0.71$ |
| $\langle$QED$\rangle$ | $0.599 \pm 0.007$ | $0.641 \pm 0.001$ | $+6.17$ | $0.643 \pm 0.001$ | $+6.55$ |
| $\langle$Weight$\rangle$ | $203.9 \pm 10.6$ | $294.8 \pm 0.8$ | $-8.56$ | $261.4 \pm 0.8$ | $-5.42$ |
| $\langle Z_0 \rangle$ | Reference | – | -1.00 | – | -0.26 |

**Table 8.** Tuned classical GAN vs QGANs with $n_{qb} = 5$, $n_l = 2$, and dual readout.

| Metrics | Classical tuned | Styled simple QGAN | $Z_0$ | Styled BEL QGAN | $Z_0$ |
|---|---|---|---|---|---|
| $N_{params}$ | 705,162 | 40 | | 210 | |
| $\epsilon_d$ | $0.481 \pm 0.031$ | $0.526 \pm 0.003$ | $+1.46$ | $0.522 \pm 0.004$ | $+1.32$ |
| $\epsilon_v$ | $0.917 \pm 0.012$ | $0.830 \pm 0.002$ | $-7.24$ | $0.876 \pm 0.004$ | $-3.36$ |
| $\epsilon_u$ | $0.986 \pm 0.003$ | $0.994 \pm 0.000$ | $+3.42$ | $0.993 \pm 0.001$ | $+2.80$ |
| Novelty | $0.536 \pm 0.015$ | $0.577 \pm 0.003$ | $+2.72$ | $0.548 \pm 0.004$ | $+0.76$ |
| IntDiv | $0.898 \pm 0.004$ | $0.882 \pm 0.000$ | $-4.66$ | $0.883 \pm 0.000$ | $-4.21$ |
| Filters | $0.721 \pm 0.011$ | $0.740 \pm 0.003$ | $+1.64$ | $0.719 \pm 0.003$ | $-0.12$ |
| $\epsilon_{LogP}$ | $0.898 \pm 0.007$ | $0.882 \pm 0.003$ | $-2.29$ | $0.896 \pm 0.002$ | $-0.36$ |
| $\langle$SA$\rangle$ | $2.391 \pm 0.080$ | $2.587 \pm 0.007$ | $-2.45$ | $2.450 \pm 0.006$ | $-0.74$ |
| $\langle$QED$\rangle$ | $0.599 \pm 0.007$ | $0.641 \pm 0.001$ | $+6.11$ | $0.644 \pm 0.001$ | $+6.61$ |
| $\langle$Weight$\rangle$ | $203.9 \pm 10.6$ | $297.4 \pm 0.6$ | $-8.82$ | $261.2 \pm 0.5$ | $-5.41$ |
| $\langle Z_0 \rangle$ | Reference | – | $-1.01$ | – | $-0.27$ |

**Table 9.** Tuned classical GAN vs QGANs with $n_{qb} = 5$, $n_l = 4$, and dual readout.

| Metrics | Classical tuned | Styled simple QGAN | $Z_0$ | Styled BEL QGAN | $Z_0$ |
|---|---|---|---|---|---|
| $N_{params}$ | 716,692 | 40 | | 220 | |
| $\epsilon_d$ | $0.481 \pm 0.031$ | $0.514 \pm 0.004$ | $+1.06$ | $0.514 \pm 0.002$ | $+1.06$ |
| $\epsilon_v$ | $0.917 \pm 0.012$ | $0.833 \pm 0.003$ | $-6.99$ | $0.887 \pm 0.003$ | $-2.45$ |
| $\epsilon_u$ | $0.986 \pm 0.003$ | $0.994 \pm 0.000$ | $+3.27$ | $0.992 \pm 0.001$ | $+2.45$ |
| Novelty | $0.536 \pm 0.015$ | $0.586 \pm 0.003$ | $+3.34$ | $0.548 \pm 0.002$ | $+0.82$ |
| IntDiv | $0.898 \pm 0.004$ | $0.882 \pm 0.000$ | $-4.68$ | $0.884 \pm 0.000$ | $-3.99$ |
| Filters | $0.721 \pm 0.011$ | $0.737 \pm 0.003$ | $+1.40$ | $0.712 \pm 0.004$ | $-0.70$ |
| $\epsilon_{LogP}$ | $0.898 \pm 0.007$ | $0.885 \pm 0.002$ | $-1.91$ | $0.900 \pm 0.001$ | $+0.30$ |
| $\langle$SA$\rangle$ | $2.391 \pm 0.080$ | $2.569 \pm 0.005$ | $-2.23$ | $2.415 \pm 0.007$ | $-0.30$ |
| $\langle$QED$\rangle$ | $0.599 \pm 0.007$ | $0.645 \pm 0.001$ | $+6.81$ | $0.641 \pm 0.001$ | $+6.19$ |
| $\langle$Weight$\rangle$ | $203.9 \pm 10.6$ | $293.6 \pm 1.1$ | $-8.43$ | $250.1 \pm 0.9$ | $-4.36$ |
| $\langle Z_0 \rangle$ | Reference | – | $-0.84$ | – | $-0.10$ |

**Table 10.** Tuned classical GAN vs QGANs with $n_{qb} = 10$, $n_l = 2$, and single readout.

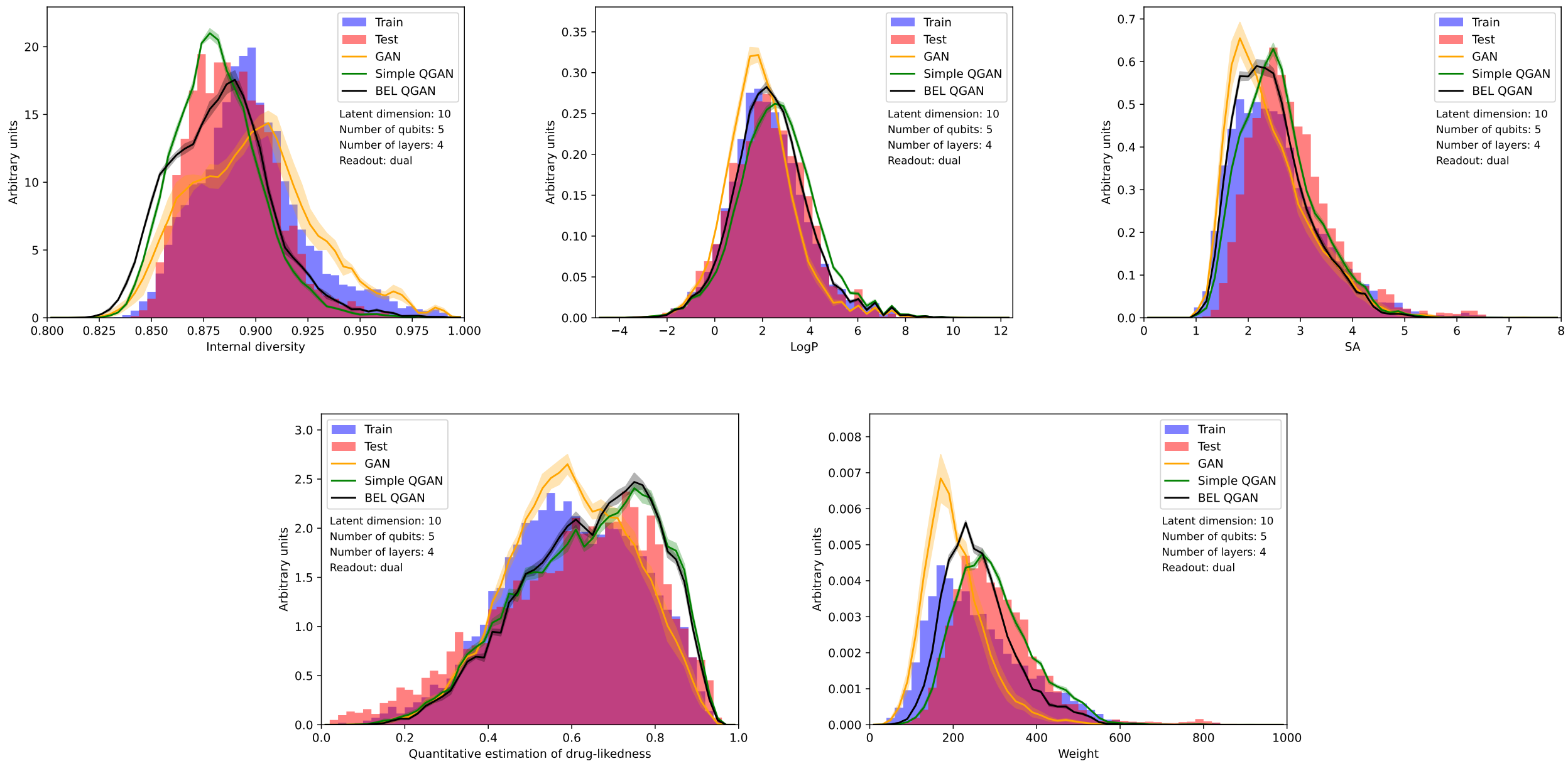


**Figure 10.** Metrics distributions for classical and quantum models. All GANs are trained with a latent dimension of 10. The quantum GANs use $n_{qb} = 5$, $n_l = 4$, and dual readout.

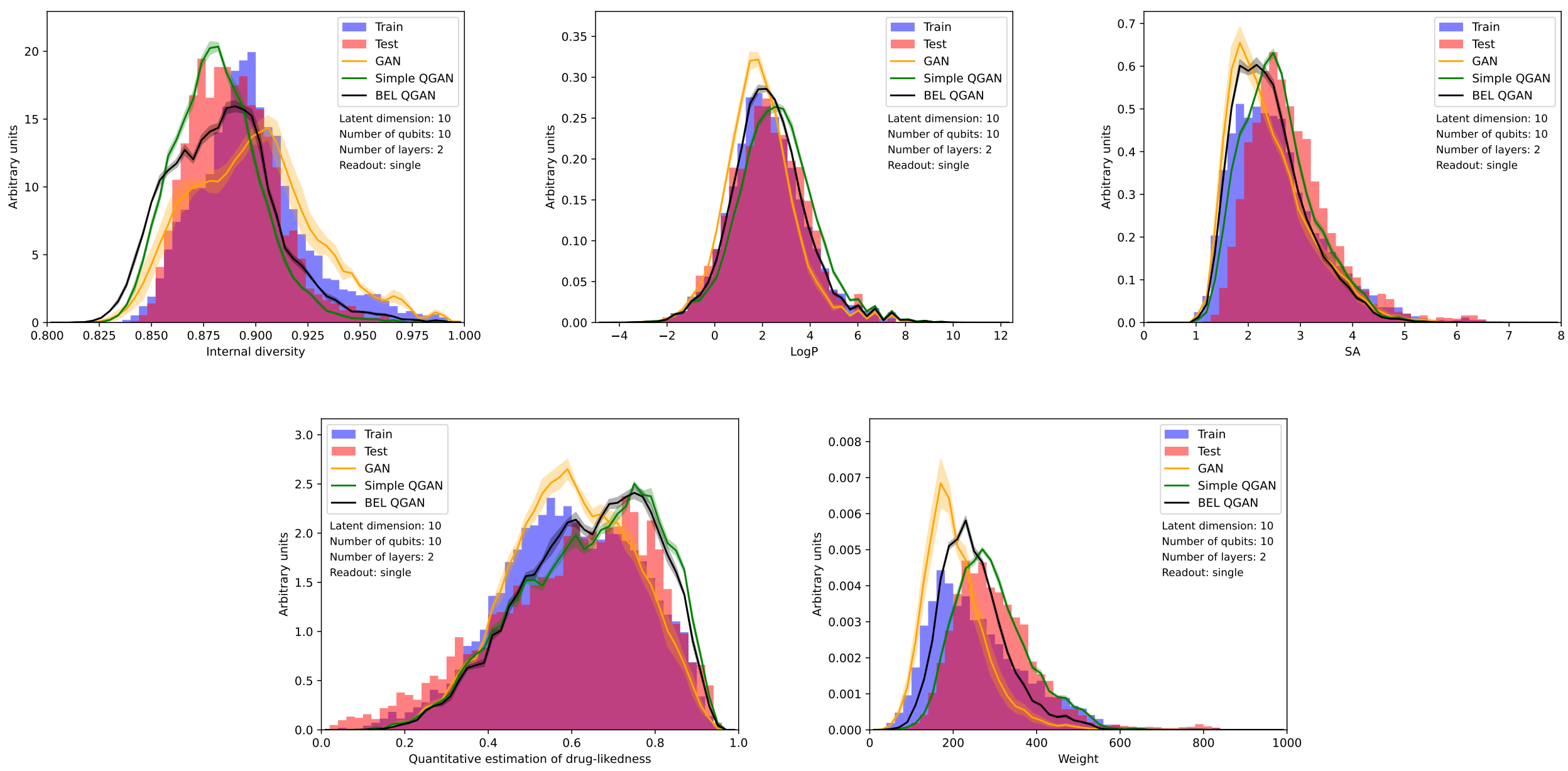


**Figure 11.** Metrics distributions for classical and quantum models. All GANs are trained with a latent dimension of 10. The quantum GANs use $n_{qb} = 10$, $n_l = 2$, and single readout.

| Metrics | Classical tuned | Styled simple QGAN | $Z_0$ | Styled BEL QGAN | $Z_0$ | styled BEL | $Z_0$ |
|---|---|---|---|---|---|---|---|
| $n_l$ | - | 4 | | 4 | | 6 | |
| $N_{params}$ | 705,162 | 80 | | 420 | | 620 | |
| $\epsilon_d$ | $0.481 \pm 0.031$ | $0.515 \pm 0.005$ | $+1.08$ | $0.516 \pm 0.005$ | $+1.13$ | $0.515 \pm 0.003$ | $+1.10$ |
| $\epsilon_v$ | $0.917 \pm 0.012$ | $0.833 \pm 0.002$ | $-6.95$ | $0.887 \pm 0.003$ | $-2.42$ | $0.888 \pm 0.002$ | $-2.41$ |
| $\epsilon_u$ | $0.986 \pm 0.003$ | $0.994 \pm 0.000$ | $+3.26$ | $0.992 \pm 0.001$ | $+2.48$ | $0.992 \pm 0.001$ | $+2.62$ |
| Novelty | $0.536 \pm 0.015$ | $0.586 \pm 0.002$ | $+3.37$ | $0.549 \pm 0.002$ | $+0.87$ | $0.550 \pm 0.001$ | $+0.93$ |
| IntDiv | $0.898 \pm 0.004$ | $0.882 \pm 0.000$ | $-4.69$ | $0.884 \pm 0.000$ | $-4.01$ | $0.884 \pm 0.000$ | $-3.99$ |
| Filters | $0.721 \pm 0.011$ | $0.737 \pm 0.003$ | $+1.41$ | $0.714 \pm 0.002$ | $-0.56$ | $0.712 \pm 0.003$ | $-0.77$ |
| $\epsilon_{LogP}$ | $0.898 \pm 0.007$ | $0.885 \pm 0.003$ | $-1.87$ | $0.900 \pm 0.002$ | $+0.25$ | $0.897 \pm 0.002$ | $-0.11$ |
| $\langle$SA$\rangle$ | $2.391 \pm 0.080$ | $2.571 \pm 0.007$ | $-2.25$ | $2.417 \pm 0.011$ | $-0.32$ | $2.421 \pm 0.004$ | $-0.37$ |
| $\langle$QED$\rangle$ | $0.599 \pm 0.007$ | $0.645 \pm 0.001$ | $+6.83$ | $0.641 \pm 0.001$ | $+6.15$ | $0.641 \pm 0.001$ | $+6.23$ |
| $\langle$Weight$\rangle$ | $203.9 \pm 10.6$ | $293.7 \pm 1.2$ | $-8.44$ | $251.1 \pm 1.9$ | $-4.39$ | $249.9 \pm 0.9$ | $-4.33$ |
| $\langle Z_0 \rangle$ | Reference | – | -0.83 | – | -0.08 | - | -0.11 |

**Table 11.** Tuned classical GAN vs QGANs with $n_{qb} = 10$, $n_l = 4, 6$, and single readout.

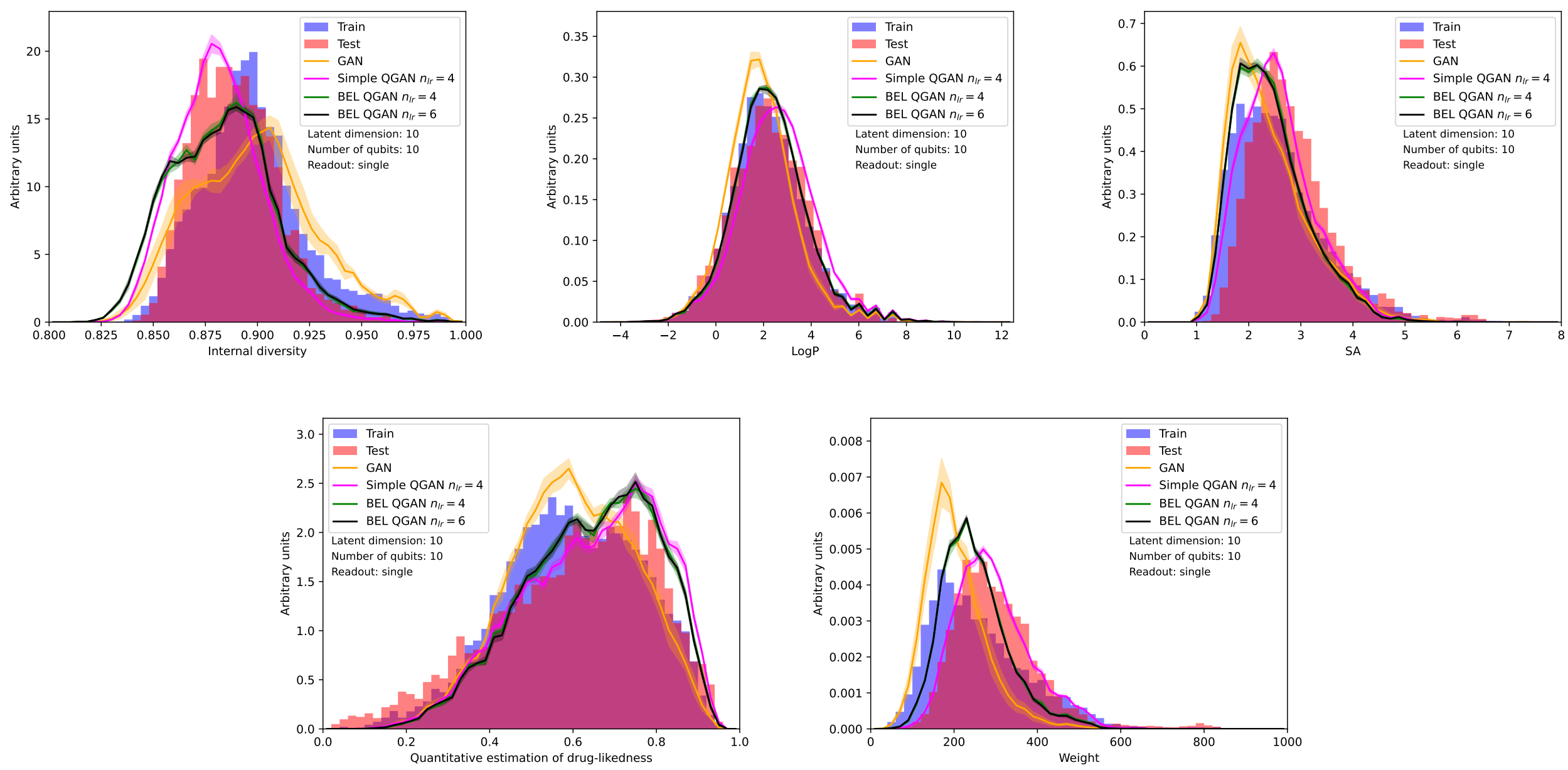


**Figure 12.** Metrics distributions for classical and quantum models. All GANs are trained with a latent dimension of 10. The quantum GANs use $n_{qb} = 10$, $n_l = 4$ and 6, and single readout.

| Metrics | Classical tuned | Styled simple QGAN | $Z_0$ | Styled BEL QGAN | $Z_0$ |
|---|---|---|---|---|---|
| **Latent dimension** | 20 | 20 | | 20 | |
| $N_{params}$ | 716,692 | 40 | | 220 | |
| $\epsilon_d$ | $0.526 \pm 0.022$ | $0.507 \pm 0.003$ | $-0.85$ | $0.519 \pm 0.005$ | $-0.32$ |
| $\epsilon_v$ | $0.901 \pm 0.008$ | $0.911 \pm 0.004$ | $+1.23$ | $0.910 \pm 0.002$ | $+1.22$ |
| $\epsilon_u$ | $0.990 \pm 0.002$ | $0.991 \pm 0.001$ | $+0.25$ | $0.991 \pm 0.001$ | $+0.05$ |
| Novelty | $0.488 \pm 0.018$ | $0.484 \pm 0.001$ | $-0.22$ | $0.484 \pm 0.002$ | $-0.25$ |
| IntDiv | $0.893 \pm 0.002$ | $0.890 \pm 0.000$ | $-1.42$ | $0.892 \pm 0.000$ | $-0.64$ |
| Filters | $0.724 \pm 0.004$ | $0.720 \pm 0.002$ | $-0.88$ | $0.722 \pm 0.003$ | $-0.56$ |
| $\epsilon_{LogP}$ | $0.897 \pm 0.004$ | $0.900 \pm 0.002$ | $+0.69$ | $0.897 \pm 0.003$ | $+0.11$ |
| $\langle$SA$\rangle$ | $2.446 \pm 0.034$ | $2.393 \pm 0.004$ | $+1.56$ | $2.421 \pm 0.003$ | $+0.75$ |
| $\langle$QED$\rangle$ | $0.613 \pm 0.006$ | $0.620 \pm 0.001$ | $+1.24$ | $0.618 \pm 0.001$ | $+0.93$ |
| $\langle$Weight$\rangle$ | $234.4 \pm 11.7$ | $231.7 \pm 0.9$ | $+0.23$ | $233.4 \pm 0.7$ | $+0.09$ |
| $\langle Z_0 \rangle$ | Reference | – | +0.18 | – | +0.14 |

**Table 12.** Quantum GANs with latent dimension of 20, $n_{qb} = 10$, $n_l = 2$, and dual readout.

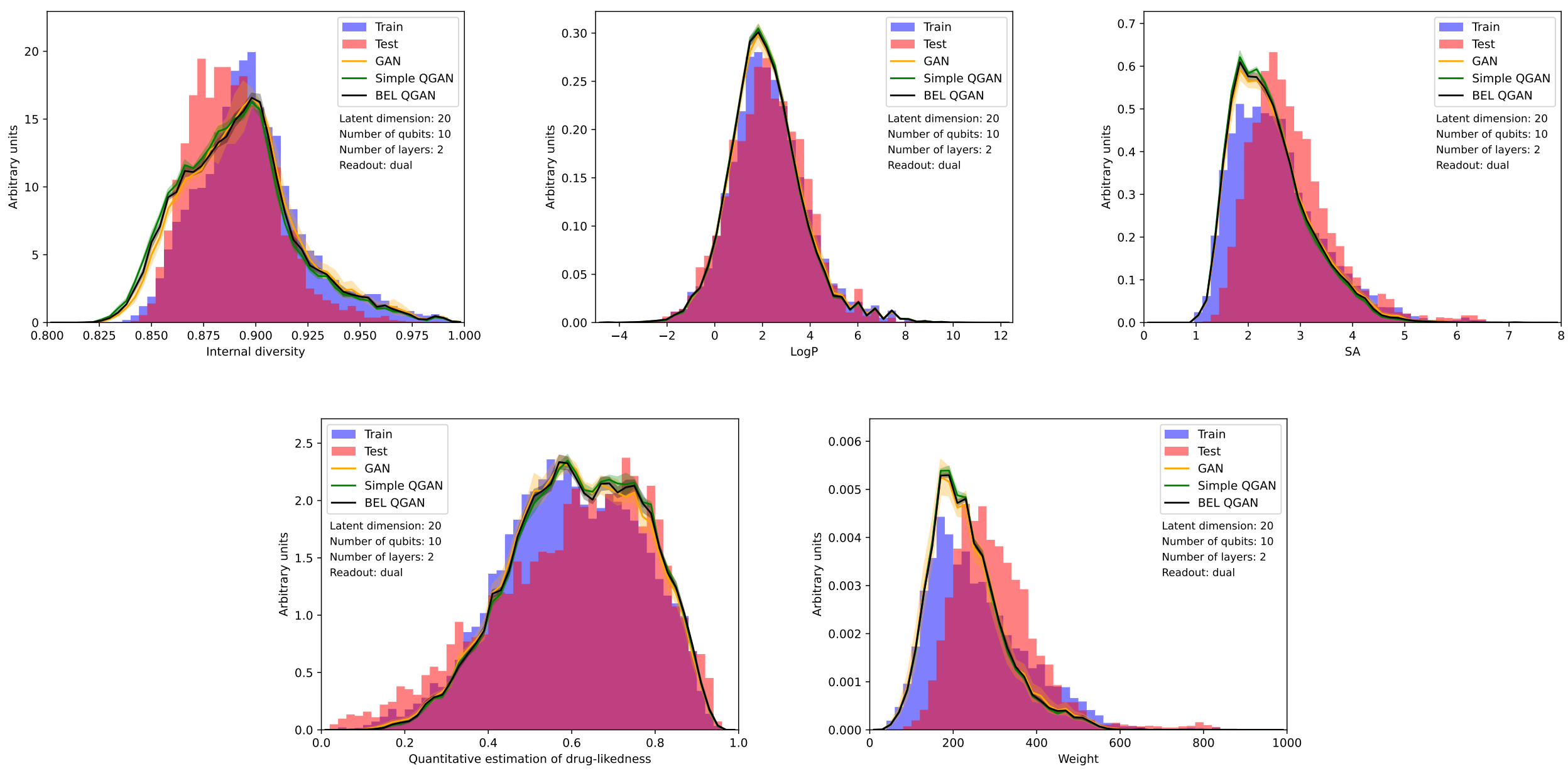


**Figure 13.** Metrics distributions for classical and quantum models. All GANs are trained with a latent dimension of 20. The quantum GANs use $n_{qb} = 10$, $n_l = 2$, and dual readout.

| Metrics | Classical tuned | Styled simple QGAN | $Z_0$ | Styled BEL QGAN | $Z_0$ |
|---|---|---|---|---|---|
| **Latent dimension** | 30 | 30 | | 30 | |
| $N_{params}$ | 728,222 | 60 | | 330 | |
| $\epsilon_d$ | $0.500 \pm 0.066$ | $0.496 \pm 0.003$ | $-0.06$ | $0.504 \pm 0.002$ | $+0.07$ |
| $\epsilon_v$ | $0.884 \pm 0.032$ | $0.895 \pm 0.002$ | $+0.32$ | $0.903 \pm 0.003$ | $+0.57$ |
| $\epsilon_u$ | $0.990 \pm 0.004$ | $0.992 \pm 0.001$ | $+0.42$ | $0.991 \pm 0.001$ | $+0.39$ |
| Novelty | $0.504 \pm 0.018$ | $0.476 \pm 0.003$ | $-1.55$ | $0.471 \pm 0.003$ | $-1.81$ |
| IntDiv | $0.896 \pm 0.003$ | $0.888 \pm 0.000$ | $-2.57$ | $0.891 \pm 0.000$ | $-1.75$ |
| Filters | $0.719 \pm 0.015$ | $0.725 \pm 0.002$ | $+0.38$ | $0.723 \pm 0.003$ | $+0.25$ |
| $\epsilon_{LogP}$ | $0.894 \pm 0.012$ | $0.901 \pm 0.002$ | $+0.55$ | $0.900 \pm 0.002$ | $+0.44$ |
| $\langle$SA$\rangle$ | $2.451 \pm 0.144$ | $2.349 \pm 0.002$ | $+0.71$ | $2.367 \pm 0.005$ | $+0.59$ |
| $\langle$QED$\rangle$ | $0.604 \pm 0.006$ | $0.624 \pm 0.001$ | $+3.23$ | $0.619 \pm 0.001$ | $+2.34$ |
| $\langle$Weight$\rangle$ | $225.8 \pm 30.1$ | $232.8 \pm 0.5$ | $-0.23$ | $226.8 \pm 0.9$ | $-0.03$ |
| $\langle Z_0 \rangle$ | Reference | – | +0.12 | – | +0.11 |

**Table 13.** Quantum GANs with a latent dimension of 30, $n_{qb} = 15$, $n_l = 2$, and dual readout.

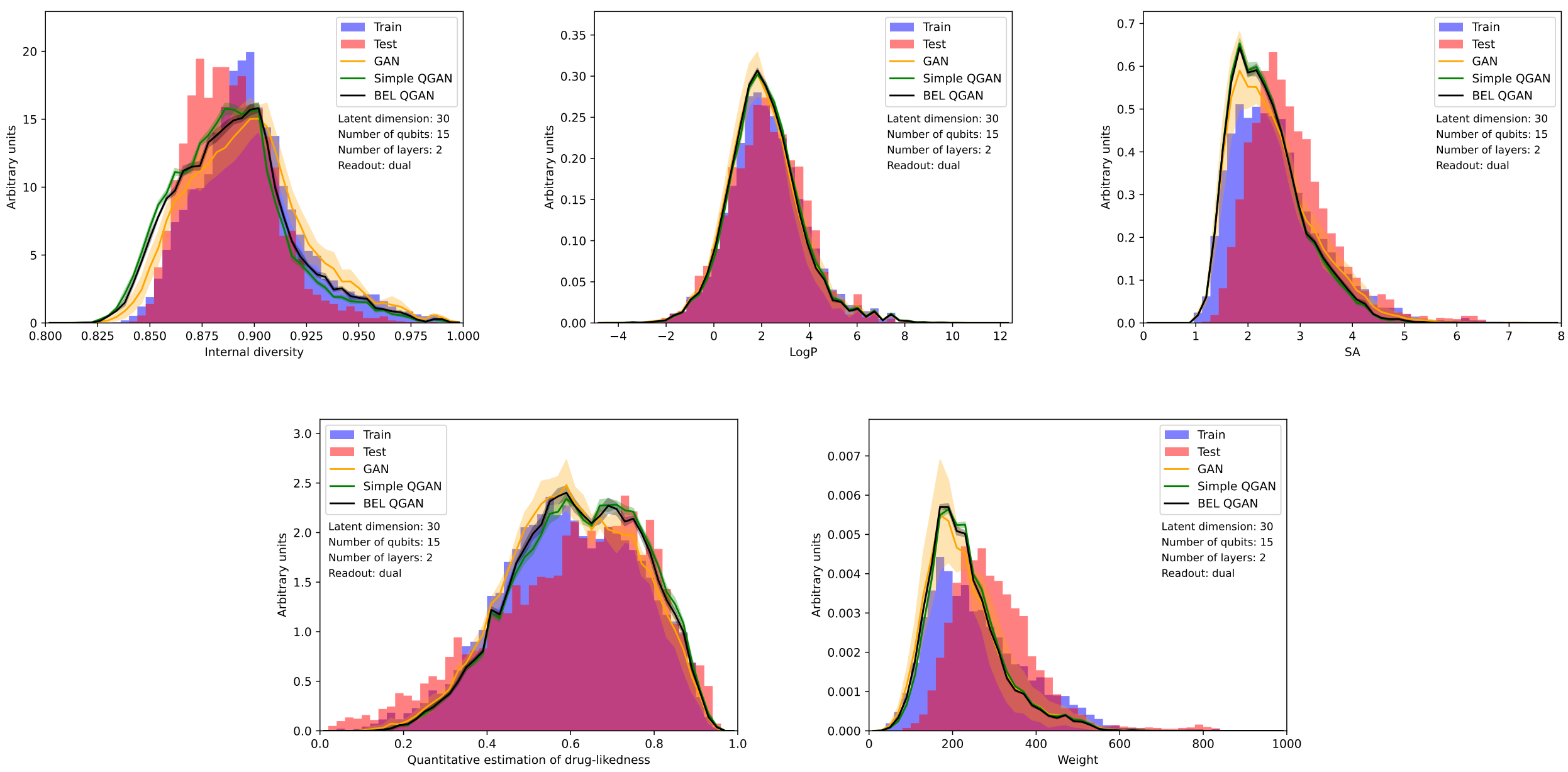


**Figure 14.** Metrics distributions for classical and quantum models. All GANs are trained with a latent dimension of 30. The quantum GANs use $n_{qb} = 15$, $n_l = 2$, and dual readout.

| Latent Dimension | 10 | 20 | $Z_0$ | 30 | $Z_0$ |
|---|---|---|---|---|---|
| $N_{params}$ | $705,162$ | $716,692$ | | $728,222$ | |
| $\epsilon_d$ | $0.481 \pm 0.031$ | $0.526 \pm 0.022$ | $+1.19$ | $0.500 \pm 0.066$ | $+0.25$ |
| $\epsilon_v$ | $0.917 \pm 0.012$ | $0.901 \pm 0.008$ | $-1.16$ | $0.884 \pm 0.032$ | $-0.96$ |
| $\epsilon_u$ | $0.986 \pm 0.003$ | $0.990 \pm 0.002$ | $+1.59$ | $0.990 \pm 0.004$ | $+0.84$ |
| Novelty | $0.536 \pm 0.015$ | $0.488 \pm 0.018$ | $-2.04$ | $0.504 \pm 0.018$ | $-1.39$ |
| IntDiv | $0.898 \pm 0.004$ | $0.893 \pm 0.002$ | $-1.19$ | $0.896 \pm 0.003$ | $-0.43$ |
| Filters | $0.721 \pm 0.011$ | $0.724 \pm 0.004$ | $+0.28$ | $0.719 \pm 0.015$ | $-0.07$ |
| $\epsilon_{LogP}$ | $0.898 \pm 0.007$ | $0.897 \pm 0.004$ | $-0.14$ | $0.894 \pm 0.012$ | $-0.26$ |
| $\langle$SA$\rangle$ | $2.391 \pm 0.080$ | $2.446 \pm 0.034$ | $-0.64$ | $2.451 \pm 0.144$ | $-0.36$ |
| $\langle$QED$\rangle$ | $0.599 \pm 0.007$ | $0.613 \pm 0.006$ | $+1.54$ | $0.604 \pm 0.006$ | $+0.49$ |
| $\langle$Weight$\rangle$ | $203.9 \pm 10.6$ | $234.4 \pm 11.7$ | $-1.94$ | $225.8 \pm 30.1$ | $-0.69$ |
| $\langle Z_0 \rangle$ | Reference | – | $-0.25$ | – | $-0.26$ |

**Table 14.** Classical GAN comparison of different latent dimensions.

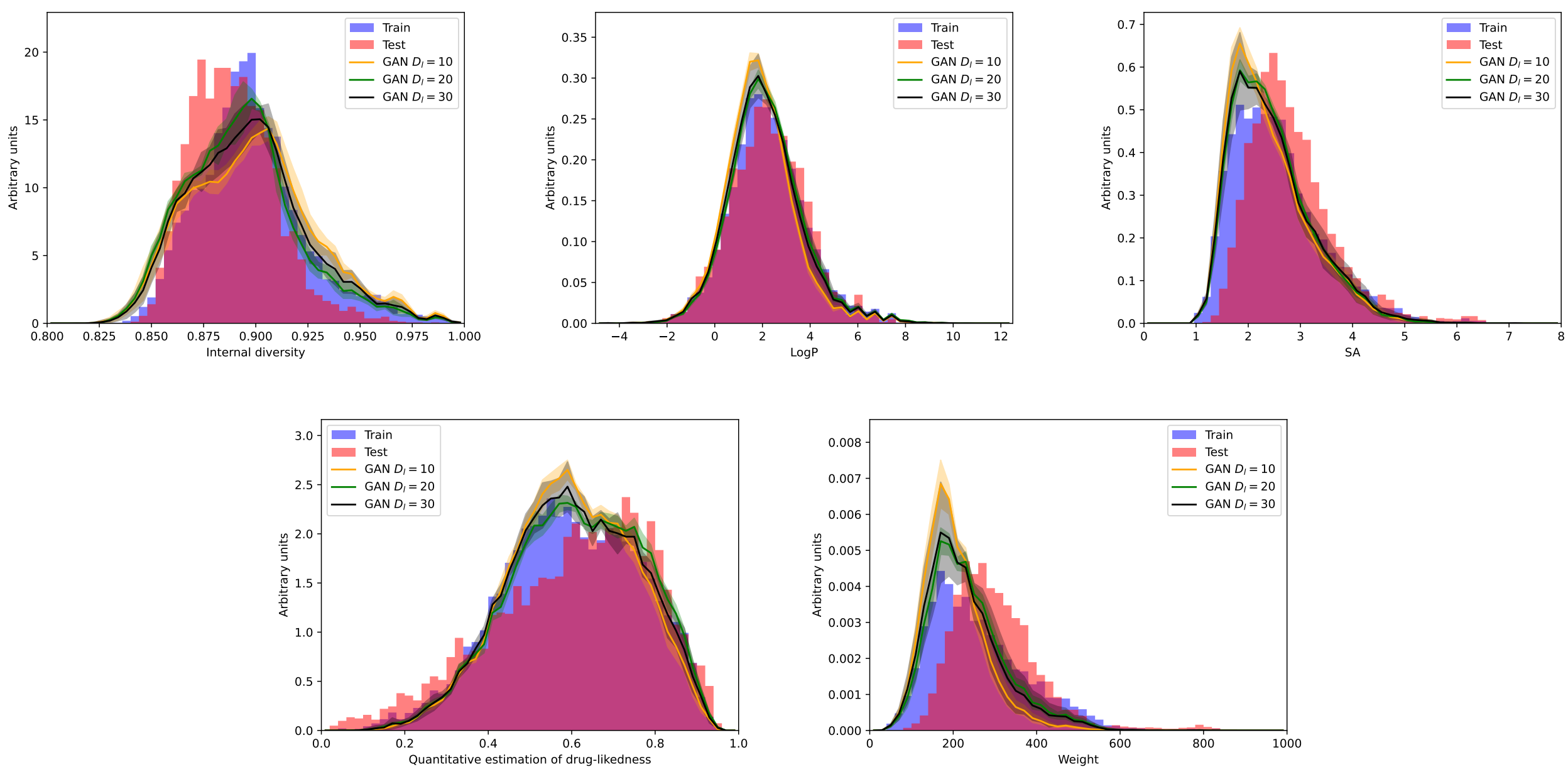


**Figure 15.** Metrics distributions for classical models trained with different latent dimensions.

# 8 SUPPLEMENTARY MATERIAL: CLASSICAL GAN STUDY WITH SAME-ORDER CAPACITY AS QGAN ARCHITECTURE

The nominal architecture and parameter settings in the main text have been taken from the MOSES package, resulting in 705,162 trainable parameters. Given that training was performed on a subset of the entire MOSES dataset, it is important to check whether a comparably smaller capacity for the classical GAN could outperform the nominal classical GAN architecture, such that more robust training would be observed and the corresponding metrics would improve significantly. We have included in the main text results from a classical latent GAN with exact matched capacity for the generator; we extend this analysis with other capacities in this section.

We have performed a supplementary control study with a downgraded architecture, such that the number of trainable parameters is roughly the same order of magnitude as the number of trainable parameters of the BEL QGAN (110 trainable parameters). As described in Section 2.2.2 of the main text, the classical generator consists of five linear layers with increasing number of nodes. Three low-parameter scenarios were defined by replacing the five-layer MOSES generator architecture $[128, 256, 512, 1024, D_l]$ by a single hidden layer followed by the output layer, $[D, D_l]$ with 1) $D = 32$ resulting in 682 parameters; 2) $D = 16$ resulting in 346 parameters; 3) $D = 10$ resulting in the exact matched capacity, 110 parameters. Two extra scenarios used a two-hidden-layer architecture, $[10, D, D_l]$, with $D = 5$ resulting in 115 parameters and $D = 4$ resulting in 94 parameters. We use the same VAE, the same discriminator, noise distribution, and tuned hyperparameters as in the main text.

We present in Figure 16 the mean values of the loss function with the uncertainty band defined as the $1\sigma$ standard deviation calculated with five runs with different seeds, as in the main text, for the single-hidden-layer scenarios with 682, 346, and 110 trainable parameters. The number of epochs where the loss function shows stability has been estimated to be $N_{ep} = 200$ for the 682-parameter scenario, $N_{ep} = 400$ for the 346-parameter scenario, and $N_{ep} = 300$ for the 110-parameter scenario and the two-hidden-layer scenarios with 115 and 94 trainable parameters. When compared with the loss function of the reference classical GAN in Figure 5 of the main text, similar behavior is obtained, and the robustness of the training is not significantly enhanced, which would have corresponded to a smoothing of the generator loss function and a much smaller band. This indicates that overfitting of the large number of trainable parameters is likely not the reason we observe a less robust training with the reference classical GAN compared to our styled BEL QGAN. We also present in Figure 17 the same analysis, but for the two-hidden-layer scenario with 115 and 94 trainable parameters. The loss curves are definitively smoother than for the nominal GAN and for the one-hidden-layer loss functions, including the matched-capacity scenario. However, they are still not as smooth as the QGAN loss functions, indicating that the QGAN architecture is more robust than the classical generators investigated in this work.

Table 15 presents the comparison and per-metric statistical compatibility between the reference GAN and the one-hidden-layer low-parameter scenarios presented in this section. While the difference is within one standard deviation, with $|\langle Z_0 \rangle| \leq 1$, we observe a consistent trend toward negative $Z_0$ across all drug-relevant metrics in the three scenarios, including the exact matched capacity scenario compared to BEL QGAN: this indicates a consistent slight degradation of performance. This contrasts with the results we have obtained with the styled QGAN in the main text, which displayed statistically significant improvement in some of the individual metrics. We display in Table 16 the corresponding comparison, but against the two-hidden-layer scenarios with 115 and 94 trainable parameters. There is still an overall statistical compatibility with the nominal reference scenario (within one standard deviation), but the situation slightly improves overall for the 94-parameter scenario with $\langle Z_0 \rangle = +0.16$. Interestingly, the drug-efficient metrics are now slightly better than for the nominal scenario, in contrast with the previous single-hidden-layer scenarios. However, not a single individual metric yields a significant improvement over the reference MOSES baseline, contrary to what we observe with the BEL QGAN.

We present in Figure 18 and Figure 19 the distributions of the metrics themselves for the samples generated by the five classical GANs. While the results are statistically consistent, there is a slight observable difference between the reference and the low-parameter scenarios.

In conclusion, this test shows that the reference GAN with architecture taken from MOSES does not suffer from performance degradation due to the large number of parameters. In contrast, single-hidden-layer classical GANs with a number of trainable parameters roughly matching the number of trainable

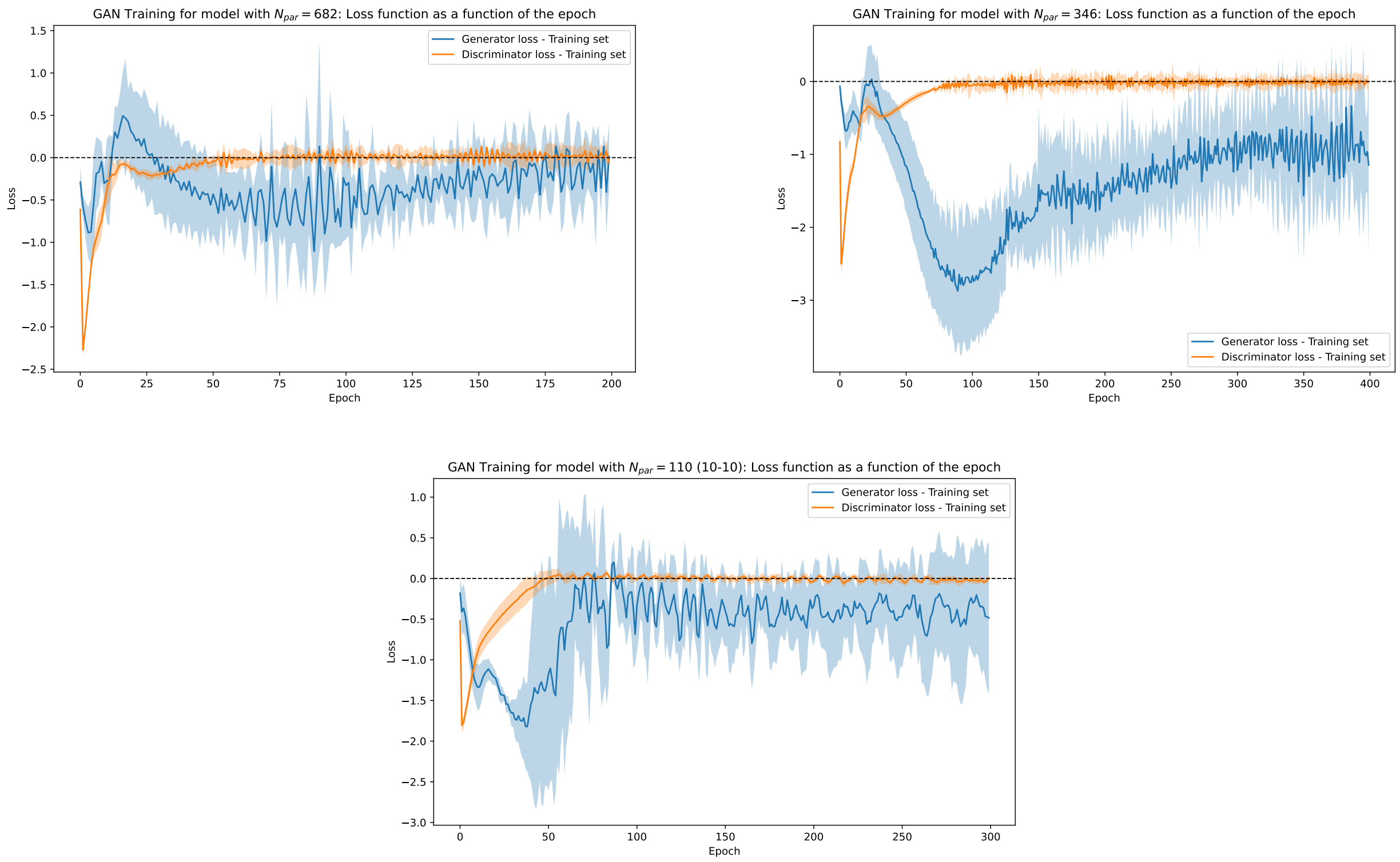


**Figure 16.** Loss functions for the low parametric GANs with 682 (upper left), 346 (upper right), and 110 (lower row) trainable parameters. The number of epochs is chosen so that the three loss functions are stable. Each model was run with five different seeds, and the shaded band is defined as $\pm 1\sigma$ deviation.

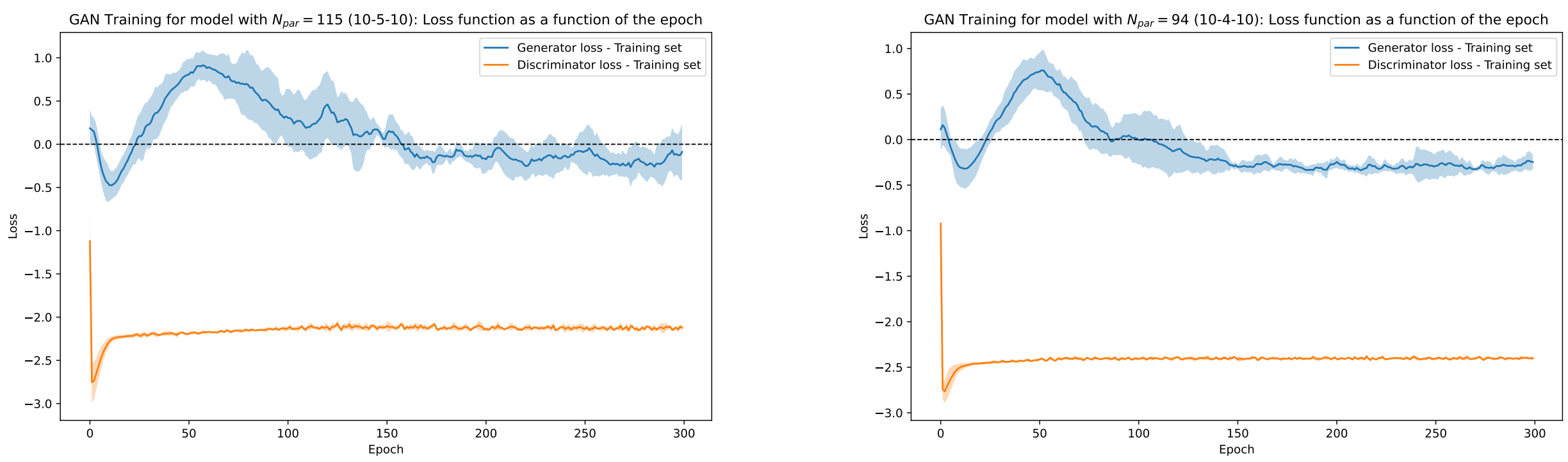


**Figure 17.** Same as Figure 16 but for two-hidden-layer scenarios with 115 (left panel) and 94 (right panel) trainable parameters.

parameters of the styled BEL QGAN slightly underperform for most of the metrics compared to the reference classical GAN in the main text. Only the 94-parameter scenario with two hidden layers slightly overperforms, but it still does not provide with any individual metric significantly improving over the nominal MOSES baseline, contrary to our proposed BEL QGAN.

| $N_{params}$ | 705, 162 | 682 | $Z_0$ | 346 | $Z_0$ | 110 | $Z_0$ |
|---|---|---|---|---|---|---|---|
| $\epsilon_d$ | 0.481 ± 0.031 | 0.519 ± 0.006 | +1.21 | 0.522 ± 0.005 | +1.33 | 0.523 ± 0.001 | +1.36 |
| $\epsilon_v$ | 0.917 ± 0.012 | 0.910 ± 0.003 | -0.57 | 0.912 ± 0.003 | -0.41 | 0.910 ± 0.001 | -0.56 |
| $\epsilon_u$ | 0.986 ± 0.003 | 0.985 ± 0.001 | -0.27 | 0.985 ± 0.001 | -0.04 | 0.986 ± 0.001 | +0.00 |
| Novelty | 0.536 ± 0.015 | 0.496 ± 0.004 | -2.67 | 0.498 ± 0.004 | -2.52 | 0.495 ± 0.003 | -2.79 |
| IntDiv | 0.898 ± 0.004 | 0.902 ± 0.000 | +1.05 | 0.902 ± 0.000 | +0.91 | 0.902 ± 0.000 | +0.97 |
| Filters | 0.721 ± 0.011 | 0.721 ± 0.002 | +0.03 | 0.722 ± 0.002 | +0.11 | 0.722 ± 0.003 | +0.09 |
| $\epsilon_{LogP}$ | 0.898 ± 0.007 | 0.894 ± 0.002 | -0.57 | 0.895 ± 0.001 | -0.50 | 0.895 ± 0.002 | -0.37 |
| ⟨SA⟩ | 2.391 ± 0.080 | 2.473 ± 0.006 | -1.03 | 2.462 ± 0.007 | -0.89 | 2.472 ± 0.005 | -1.02 |
| ⟨QED⟩ | 0.599 ± 0.007 | 0.594 ± 0.001 | -0.74 | 0.595 ± 0.002 | -0.59 | 0.594 ± 0.001 | -0.73 |
| ⟨Weight⟩ | 203.9 ± 10.6 | 214.2 ± 2.1 | -0.96 | 213.9 ± 1.1 | -0.94 | 215.2 ± 1.0 | -1.07 |
| $\langle Z_0\rangle$ | Reference | – | $-0.45$ | – | $-0.35$ | – | -0.41 |

**Table 15.** Metrics comparison for GANs trained with different number of generator trainable parameters. Each model was run with five different seeds to calculate the $\pm 1\sigma$ deviation on the metrics.

| $N_{params}$ | 705, 162 | 115 | $Z_0$ | 94 | $Z_0$ |
|---|---|---|---|---|---|
| $\epsilon_d$ | 0.481 ± 0.031 | 0.491 ± 0.025 | +0.23 | 0.473 ± 0.013 | -0.26 |
| $\epsilon_v$ | 0.917 ± 0.012 | 0.920 ± 0.009 | +0.17 | 0.924 ± 0.002 | +0.55 |
| $\epsilon_u$ | 0.986 ± 0.003 | 0.986 ± 0.002 | +0.27 | 0.986 ± 0.002 | +0.26 |
| Novelty | 0.536 ± 0.015 | 0.518 ± 0.013 | -0.90 | 0.529 ± 0.003 | -0.47 |
| IntDiv | 0.898 ± 0.004 | 0.898 ± 0.002 | -0.19 | 0.898 ± 0.002 | -0.19 |
| Filters | 0.721 ± 0.011 | 0.723 ± 0.003 | +0.23 | 0.725 ± 0.004 | +0.38 |
| $\epsilon_{LogP}$ | 0.898 ± 0.007 | 0.898 ± 0.004 | +0.00 | 0.901 ± 0.004 | +0.47 |
| ⟨SA⟩ | 2.391 ± 0.080 | 2.389 ± 0.046 | +0.02 | 2.359 ± 0.025 | +0.39 |
| ⟨QED⟩ | 0.599 ± 0.007 | 0.601 ± 0.005 | +0.23 | 0.600 ± 0.005 | +0.08 |
| ⟨Weight⟩ | 203.9 ± 10.6 | 206.5 ± 10.5 | -0.17 | 199.7 ± 3.5 | +0.38 |
| $\langle Z_0\rangle$ | Reference | – | $-0.01$ | – | $+0.16$ |

**Table 16.** Same as in Table 15 for the two-hidden-layer scenarios with 115 and 94 trainable parameters.

## 9 SUPPLEMENTARY MATERIAL: QUANTUM HARDWARE CHARACTERIZATION, NOISY SIMULATION, AND LATENT-VECTOR DISTRIBUTIONS

This section documents the quantum hardware and the execution settings used for the inference-only run reported in Section 3.3 of the main text, in which 2,500 molecules have been sampled from the simulator-trained QGAN on the ibm_kingston device. No training was performed on hardware. Table 17 summarizes the model and execution configuration, Table 18 reports representative device calibration properties, and Table 19 gives the per-metric $Z_0$ significance of the hardware run relative to the noiseless simulation, see eq 5 of the main text, calculated with bootstrap standard errors. We also report in Table 20 a matched noise-model simulation on the same trained generator, and in Figure 20 and Table 21 a comparison of the latent-vector distributions for the matched noiseless simulation, noisy simulation, and ibm_kingston hardware run, before VAE decoding. Transpiled circuit depth, two-qubit-gate count, and physical-qubit layout have been read from the executed circuits stored in the IBM Runtime job records for all 5,000 Estimator jobs (2,500 $X$/ $Z$ pairs). We observe 13 distinct logical-to-physical layout mappings and nine distinct five-qubit physical subsets across the run (the mode layout maps logical qubits 0–4 to physical indices 96, 83, 82, 76, 81 in 34% of jobs). Device-wide calibration medians in Table 18 are taken from actual calibration data for ibm_kingston (using `backend.properties(datetime=...)`) on 2025-10-04 UTC, contemporaneous with the inference session.

We have also performed a matched noisy simulation of the hardware run based on the `FakeKingston` noise model provided by IBM. We have generated 2,500 latent vectors from the same simulator-trained styled-BEL generator ($n_{qb} = 5$, $n_l = 2$, dual readout, 110 trainable parameters) using the Aer simulator

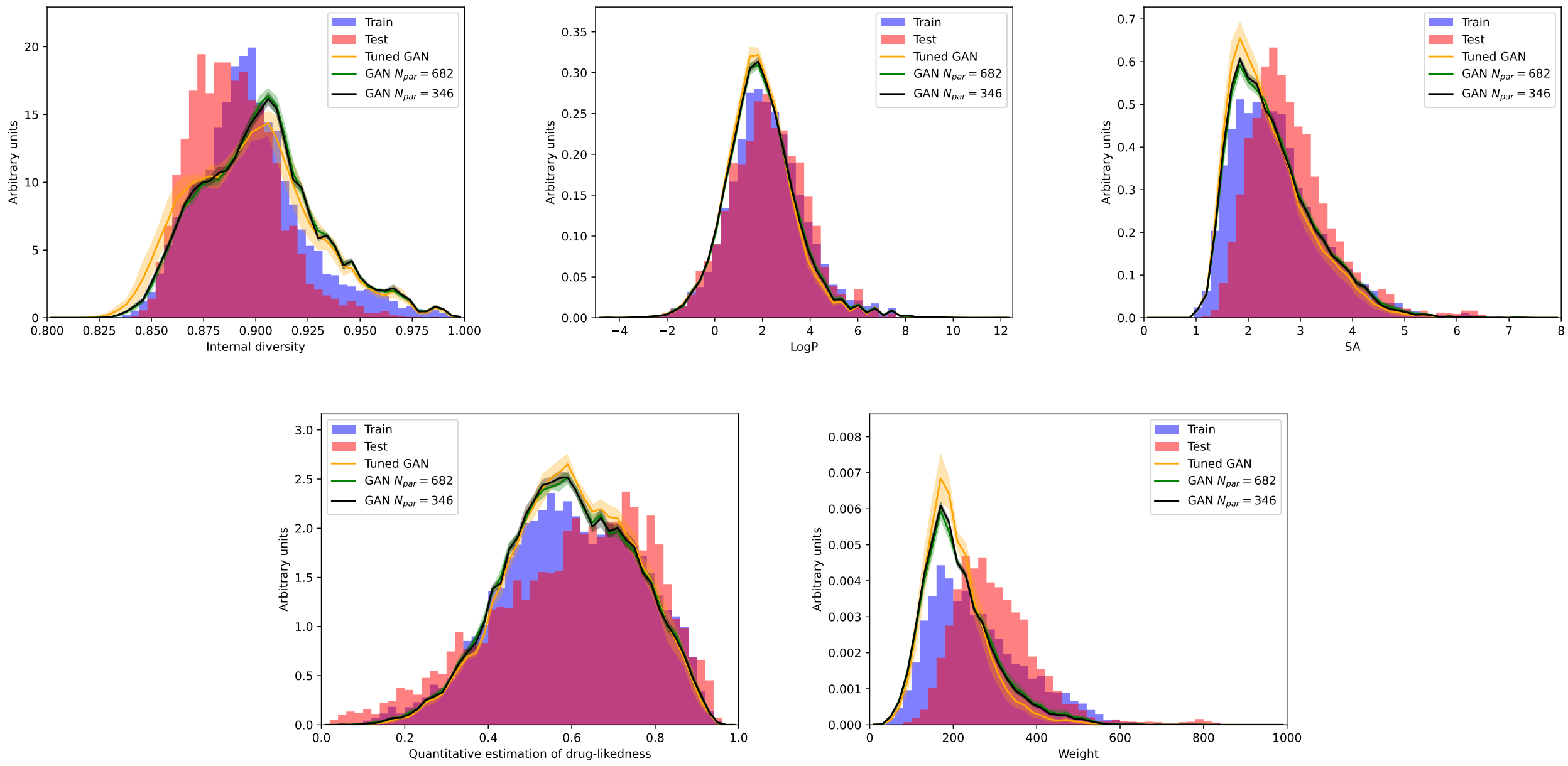


**Figure 18.** Metrics distributions for GANs trained with different number of generator trainable parameters (scenarios with 682 and 346 trainable parameters). Each model was run with five different seeds, and the shaded band is defined as the $\pm 1\sigma$ deviation.

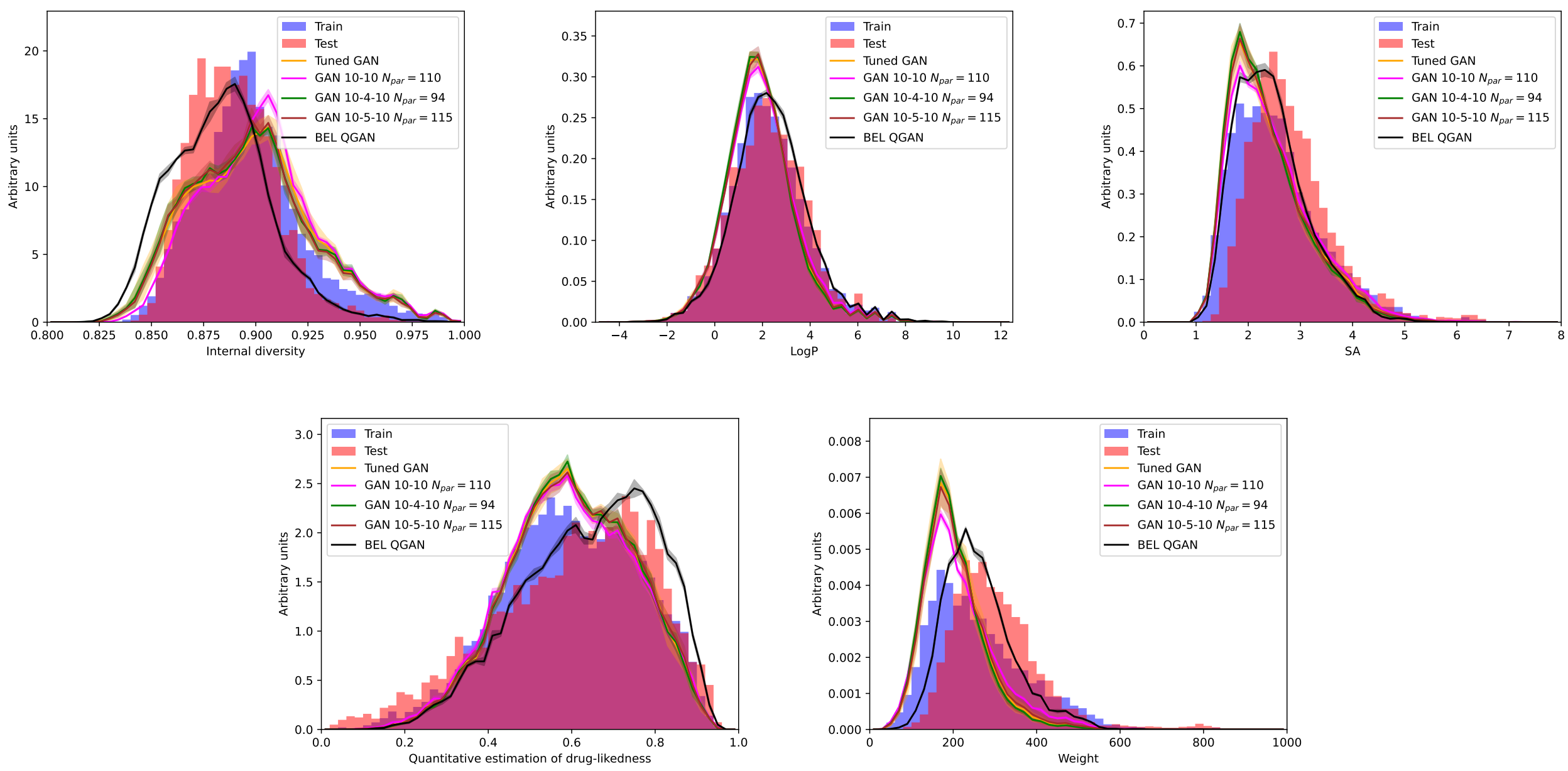


**Figure 19.** Same as in Figure 18 for the low-parameter scenarios with 115, 110, and 94 trainable parameters.

backend, initialized with the `FakeKingston` snapshot, using per-sample execution and 1,000 shots per circuit. The resulting latent vectors were decoded with the identical tuned VAE used in our work for the classical GAN, the noiseless-simulation QGAN run, and the actual hardware runs. We then evaluated the metrics with the same MOSES pipeline as in Table 6 of the main text, using again nonparametric bootstrap resampling, with $B = 10,000$ bootstrap resamples, to calculate the confidence intervals in Table 20. This offline simulation isolates the effect of gate, readout, and decoherence errors encoded in the device noise

| Property | Value |
|---|---|
| Quantum processor | ibm_kingston (IBM Heron r2) |
| Qubit topology | heavy-hex |
| Total physical qubits on device | 156 |
| Circuit | styled BEL |
| Qubits / readout | 5 / dual |
| Number of layers $n_l$ | 2 |
| Latent-vector dimension | 10 |
| Trainable parameters | 110 |
| Number of generated molecules | 2,500 |
| Shots per circuit | 1,000 |
| Transpilation optimization level | 2 (Qiskit default) |
| Resilience level | 0 (no error mitigation) |
| Execution mode | batch (IBM Cloud) |
| Calibration snapshot date | 2025-10-04, 17:00 UTC |
| Physical qubit indices (mode layout) | 96, 83, 82, 76, 81 |
| Distinct layouts / physical-qubit sets | 13 / 9 (over 5,000 jobs) |
| X/Z readout scheme | Separate Estimator jobs (alternating $X$ then $Z$) |
| Qiskit IBM Runtime primitive | Estimator |
| Transpiled circuit depth (median) | 171 ($X$: 171; $Z$: 171; range 147–190) |
| Transpiled two-qubit-gate count (median) | 75 CZ ($X$: 75; $Z$: 75; range 71–88) |
| Number of jobs | 5,000 |
| Execution date(s) | 2025-10-04, 14:42–22:32 UTC |
| Total QPU time | 10,377 s ($\approx$ 2.9 h) |

**Table 17.** Model and execution configuration for the illustrative inference-only run on ibm_kingston. The quantum generator uses $n_{qb} = 5$, $n_l = 2$, and dual readout to produce a latent vector of dimension 10. Transpiled depth, CZ counts, and physical-qubit layout statistics are computed over all 5,000 Estimator jobs from the transpiled circuits stored in the IBM Runtime job records. The listed physical indices are the mode logical-to-physical mapping for qubits 0–4 (34% of jobs); see text for layout variation across the run.

| Calibration metric | Value | Unit |
|---|---|---|
| Basis gates | `cz`, `id`, `rz`, `sx`, `x` | – |
| Median $T_1$ | 249 | $\mu$s |
| Median $T_2$ | 142 | $\mu$s |
| Median single-qubit ($\sqrt{X}$) gate error | $2.4 \times 10^{-4}$ | – |
| Median two-qubit (CZ) gate error | $2.0 \times 10^{-3}$ | – |
| Median readout assignment error | $8.5 \times 10^{-3}$ | – |
| Median single-qubit gate time | 32 | ns |
| Median two-qubit gate time | 68 | ns |

**Table 18.** Representative device-wide calibration properties for the IBM Heron r2 ibm_kingston device on the execution date. Medians are computed over all 156 device qubits from `backend.properties(datetime=2025-10-04)`, with snapshot taken at 17:00 UTC on 2025-10-04. The five physical qubits listed in Table 17 are a subset of the device qubits. Note that only the default basis gates are reported here to match the transpilation setup of the hardware run.

model, without transpilation onto a specific physical layout or the separate alternating $X$/ $Z$ Estimator job structure used on hardware.

The results in Table 20 show that the noisy simulation is in good agreement with the noiseless simulation on most chemically relevant metrics, for example with $\langle \text{QED} \rangle = 0.642$ (0.647 in the noiseless simulation) or Novelty = $0.337$ (0.338 in the noiseless simulation). Some metrics do differ beyond the bootstrap

| Metrics | QGAN (noiseless) | QGAN (ibm_kingston) | $Z_0$ |
|---|---|---|---|
| Novelty | $0.338 \pm 0.010$ | $0.341 \pm 0.011$ | $+0.18$ |
| IntDiv | $0.881 \pm 0.001$ | $0.882 \pm 0.001$ | $+0.85$ |
| Filters | $0.706 \pm 0.010$ | $0.713 \pm 0.010$ | $+0.49$ |
| $\epsilon_{LogP}$ | $0.899 \pm 0.007$ | $0.889 \pm 0.007$ | $-1.04$ |
| $\langle$SA$\rangle$ | $2.424 \pm 0.015$ | $2.485 \pm 0.016$ | $-2.81$ |
| $\langle$QED$\rangle$ | $0.647 \pm 0.003$ | $0.649 \pm 0.004$ | $+0.28$ |
| $\langle$Weight$\rangle$ | $264.8 \pm 1.8$ | $273.6 \pm 1.8$ | $-3.43$ |
| $\langle Z_0 \rangle$ | Reference | – | $-0.78$ |

**Table 19.** Per-metric $Z_0$ significance (eq 5 of the main text) for the inference-only hardware run versus the noiseless simulation, using the bootstrap standard errors of both runs as $\sigma_1^m$ and $\sigma_2^m$ ($B = 10,000$ resamples). The noiseless simulation is the reference; a negative $Z_0^m$ for a maximized metric indicates degradation of the hardware result at this bootstrap precision. These uncertainties quantify finite-sample precision of each single run and do not reflect run-to-run initialization spread.

precision, for example $\langle$Weight$\rangle$ $= 280.1$ (264.9 in the noiseless simulation) or $\langle$SA$\rangle$ $= 2.522$ (2.424 in the noiseless simulation), with bootstrap confidence intervals that do not overlap, but the difference is still limited. These results indicate that the static device noise model alone does not predict a large degradation relative to ideal simulation, meaning that e.g. the gate errors, readout errors, and decoherence errors captured in the noise model are not causing a significant degradation of the pipeline. Comparing the noisy simulation with the real hardware run, we find an overall good agreement on the central drug-design observables. In particular, $\langle$QED$\rangle$ $= 0.649$ on hardware agrees with $0.647$ on the noiseless simulator and $0.642$ on the noisy emulator within bootstrap precision. Modest resolved shifts appear for $\epsilon_{LogP}$, $\langle$SA$\rangle$, and $\langle$Weight$\rangle$ (Supplementary Information Table 19), without indicating a broad failure of simulator-trained parameters on current hardware. This suggests that effects beyond the snapshot noise model, such as layout-dependent transpilation or the separate-basis readout protocol, contribute to the residual noisy-simulation–hardware gap on these metrics. We do not expect the noisy simulation to bracket every metric exactly, consistent with the simplified execution model.

Taken together, gate, readout, and decoherence noise as modeled by `FakeKingston` does not lead to a collapse of the pipeline relative to the noiseless simulation, and the hardware decoded metrics remain broadly compatible with both the noiseless and noisy-emulator results at this bootstrap precision. We hence do not observe a broad failure of simulator-trained parameters on current quantum hardware.

| Metrics | QGAN (noiseless) | QGAN (noisy simulation) | QGAN (ibm_kingston) |
|---|---|---|---|
| $\epsilon_d$ | 0.892 | 0.893 | 0.891 |
| $\epsilon_v$ | 0.934 | 0.914 | 0.904 |
| $\epsilon_u$ | 1.000 | 0.999 | 0.999 |
| Novelty | 0.338 [0.318, 0.358] | 0.337 [0.316, 0.357] | 0.341 [0.320, 0.362] |
| IntDiv | 0.881 [0.879, 0.883] | 0.883 [0.881, 0.885] | 0.882 [0.880, 0.884] |
| Filters | 0.706 [0.687, 0.726] | 0.705 [0.685, 0.724] | 0.713 [0.693, 0.733] |
| $\epsilon_{LogP}$ | 0.899 [0.886, 0.913] | 0.882 [0.868, 0.896] | 0.889 [0.875, 0.902] |
| $\langle$SA$\rangle$ | 2.424 [2.395, 2.453] | 2.522 [2.492, 2.554] | 2.485 [2.455, 2.515] |
| $\langle$QED$\rangle$ | 0.647 [0.641, 0.654] | 0.642 [0.635, 0.649] | 0.649 [0.642, 0.656] |
| $\langle$Weight$\rangle$ | 264.9 [261.4, 268.3] | 280.1 [276.4, 283.9] | 273.6 [270.0, 277.2] |

**Table 20.** Metrics comparison for 2,500 molecules generated with the same simulator-trained styled-BEL QGAN ($n_{qb} = 5$, $n_l = 2$, dual readout) on the noiseless state-vector simulator, on a `FakeKingston` Aer noise-model simulator (1,000 shots), and on ibm_kingston hardware. The noisy-simulation run uses the publicly available Heron r2 device snapshot; it does not reproduce the hardware transpilation layout or the separate $X$/ $Z$ Estimator job structure. The metrics $\epsilon_d$, $\epsilon_v$, and $\epsilon_u$ are point estimates; for all other metrics, values in brackets give the $95\%$ bootstrap percentile intervals ($B = 10,000$ resamples).

We have also performed a direct comparison of the latent-vector distributions for the noiseless simulation, the noisy simulation with the `FakeKingston` noise model, and the ibm_kingston hardware run, *before* VAE decoding. We compare the 2,500 predecoder latent vectors from the three inference settings in Figure 20. For this comparison, we have generated the noiseless-simulator latent vectors by running the trained styled-BEL generator on the noiseless state-vector simulator to emit 2,500 latent vectors without outputting the corresponding molecules with the VAE decoder, using the same trained styled-BEL checkpoint as in the main text to obtain Table 6 of the main text. We have rescaled the latent vectors to the $[0, 1]$ range using the $(1 + \langle \hat{O} \rangle)/2$ formula, to match the hardware latent vectors, which are the expectation values saved from the IBM Runtime jobs and rescaled identically. The noisy-simulation latent vectors are those produced by the matched `FakeKingston` run of Table 20. Note that we have checked that the noiseless-simulator latent vectors we use for this comparison do reproduce the intended noiseless inference distribution within bootstrap precision.

We present our results in Figure 20 and Table 21. Similarly to Figure 9 of the main text, we display the per-component count histograms, including shaded uncertainty bands. In Figure 20, they correspond to the nonparametric bootstrap $95\%$ percentile interval with $B = 2,000$ resamples with replacement from the 2,500 latent values for each corresponding scenario: red lines for the noiseless simulated inference, blue lines for the noisy simulated inference using the `FakeKingston` noise model, and black lines for the hardware inference on ibm_kingston. Because the hardware inference is only performed for 2,500 samples, the binning is coarser than in Figure 9 of the main text: eight bins in Figure 20 (roughly 310 counts per bin) instead of 50 bins in Figure 9 of the main text (roughly 600 counts per bin). Note that we compare here the direct generator output rescaled in $[0, 1]$, while in the main text the comparison is in the VAE latent coordinate system, roughly $[-3, 3]$. Most components show broad overlap between the three inference scenarios (within bootstrap uncertainty bands), except for large shifts in the sixth and seventh components, as well as in the very first bin. The observed shifts for the sixth and seventh components are consistent with the anti-correlated pair discussed in Section 6 and reflect genuine hardware and shot-noise effects rather than sample-size artifact alone.

We quantify our observations in Table 21, which summarizes two-sample Kolmogorov–Smirnov (KS) statistics and one-dimensional Wasserstein ($W_1$) distances aggregated over the ten latent dimensions. All pairwise comparisons reject the null of identical marginal distributions at $p < 0.05$ for every component, reflecting finite-sample differences in means and variances. Hardware latent vectors are generally shifted toward higher values. We note that, with $N = 2,500$ samples per arm, the KS test is sensitive to the modest mean and variance shifts expected under finite-shot readout and device noise; therefore, the statistical rejection of exact equality does not by itself imply a wholesale replacement of the generator output distribution or a breakdown of the inference pipeline. Importantly, the mean $W_1$ distances remain small, with $\langle W_1 \rangle \lesssim 0.09$ for the noiseless–hardware comparison, which is consistent with the close agreement of the post-VAE drug-design metrics in Table 6 of the main text and Table 20, notably $\langle \text{QED} \rangle = 0.649$ on hardware versus $0.647$ on the noiseless simulator. The noisy simulation lies between the noiseless simulation and the hardware result on several components (notably $z_6$–$z_7$), but does not uniformly bracket hardware, in line with the simplified execution model of Table 20.

These results support the conclusion that simulator-trained parameters transfer well to real hardware in this inference run. The latent-vector histograms display a broad overlap between the noiseless simulation, noisy simulation, and hardware inference: noise affects some components, in some bins and notably for $z_6$ and $z_7$, but not enough to collapse the distribution. The mean Wasserstein distance between noiseless simulation and hardware, $\langle W_1 \rangle \simeq 0.086$, is consistent with the post-decoder drug-design metrics, for which $\langle \text{QED} \rangle$ matches the noiseless simulator within bootstrap precision and four of seven reported observables show no significant degradation ($Z_0^m \geq -1$; Table 19).

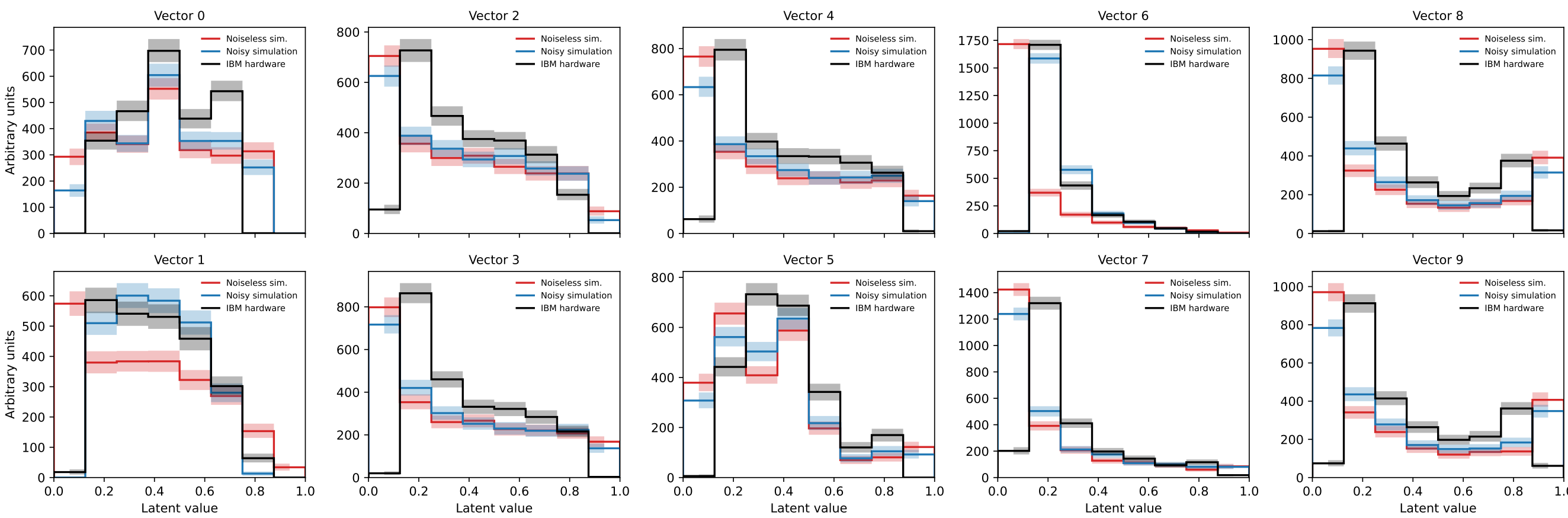


**Figure 20.** Pre-VAE latent-vector distributions on an inference with 2,500 samples from the styled-BEL QGAN ($n_{qb} = 5$, $n_l = 2$, dual readout) with a noiseless state-vector simulator (red), a `FakeKingston` Aer noisy simulation (blue), and the ibm_kingston hardware run (black). The step count histograms use eight bins, and the shaded regions display the $95\%$ bootstrap percentile bands ($B = 2,000$ resamples per scenario).

| Comparison | Mean KS | Max KS | Mean $W_1$ | Max $W_1$ |
|---|---|---|---|---|
| Noiseless sim. vs. hardware | 0.343 | 0.681 | 0.086 | 0.122 |
| Noiseless sim. vs. noisy simulation | 0.227 | 0.739 | 0.044 | 0.143 |
| Hardware vs. noisy simulation | 0.251 | 0.434 | 0.052 | 0.081 |

**Table 21.** Summary of pre-VAE latent-distribution distances for the 2,500-sample inference runs ($d_{\text{latent}} = 10$). Mean and maximum KS statistics and one-dimensional Wasserstein distances ($W_1$) are computed per latent component and aggregated over the ten latent vectors $z_0, \ldots, z_9$. The $p$-values are $< 0.05$ for all ten components in each pairwise comparison.
